# Dysfunction of exhausted T cells is enforced by MCT11-mediated lactate metabolism

**DOI:** 10.1038/s41590-024-01999-3

**Published:** 2024-11-08

**Authors:** Ronal M. Peralta, Bingxian Xie, Konstantinos Lontos, Hector Nieves-Rosado, Kellie Spahr, Supriya Joshi, B. Rhodes Ford, Kevin Quann, Andrew T. Frisch, Victoria Dean, Mary Philbin, Anthony R. Cillo, Sebastian Gingras, Amanda C. Poholek, Lawrence P. Kane, Dayana B. Rivadeneira, Greg M. Delgoffe

**Affiliations:** 1 Department of Immunology, University of Pittsburgh, Pittsburgh, PA USA; 2 Tumor Microenvironment Center, UPMC Hillman Cancer Center, Pittsburgh, PA USA; 3 Department of Medicine, Division of Hematology/Oncology, University of Pittsburgh, Pittsburgh, PA USA; 4 Department of Pediatrics, University of Pittsburgh, Pittsburgh, PA USA; 5 Center for Systems Immunology, University of Pittsburgh, Pittsburgh, PA USA

**Keywords:** Tumour immunology, Immunotherapy, Cytotoxic T cells, Cancer

## Abstract

CD8^+^ T cells are critical mediators of antitumor immunity but differentiate into a dysfunctional state, known as T cell exhaustion, after persistent T cell receptor stimulation in the tumor microenvironment (TME). Exhausted T (T_ex_) cells are characterized by upregulation of coinhibitory molecules and reduced polyfunctionality. T cells in the TME experience an immunosuppressive metabolic environment via reduced levels of nutrients and oxygen and a buildup of lactic acid. Here we show that terminally T_ex_ cells uniquely upregulate *Slc16a11*, which encodes monocarboxylate transporter 11 (MCT11). Conditional deletion of MCT11 in T cells reduced lactic acid uptake by T_ex_ cells and improved their effector function. Targeting MCT11 with an antibody reduced lactate uptake specifically in T_ex_ cells, which, when used therapeutically in tumor-bearing mice, resulted in reduced tumor growth. These data support a model in which T_ex_ cells upregulate MCT11, rendering them sensitive to lactic acid present at high levels in the TME.

## Abstract (web summary)

Here the authors show that high expression of MCT11 is key to the dysfunctionality associated with exhausted CD8^+^ T cells in tumors. By targeting MCT11, uptake of lactic acid, which is abundant in the tumor, is reduced, resulting in improved effector functions and tumor immunity.

## Main

CD8^+^ T cells have a crucial role in orchestrating immune responses against pathogens and tumors. Antigen recognition via the T cell receptor (TCR) promotes activation and expansion of a clonal army to target infected or malignant cells^1^. Upon antigen clearance, most effectors cells die, leaving a population of memory T cells which will become reactivated upon encountering their cognate antigen^2^. However, when antigen persists and T cells are exposed to continuous TCR stimulus, as in the context of cancer, they progressively differentiate into a hypofunctional state known as T cell exhaustion^3^. At least two distinct populations have been identified in the exhaustion lineage: progenitor exhausted (T_pex_) cells and terminally differentiated exhausted (T_ex_) cells^4^. T_pex_ cells are characterized by intermediate expression of PD1 and retention of self-renewal capacities maintained via the transcription factor TCF-1 (ref. ^5^). As TCR stimulation persists, T_pex_ cells ultimately become T_ex_ cells, which are characterized by a reduction in self-renewal capacity and ability to produce effector cytokines (that is, IL-2 and TNF) and upregulation of coinhibitory molecules (that is, PD1, CTLA-4, Tim3 and Lag3)^3^. Persistent stimulation by antigen leads CD8^+^ T cells infiltrating the tumor to differentiate to T_ex_ cells, ultimately contributing to tumor immune escape.

While persistent TCR stimulus may be the main driver of T cell exhaustion, metabolic stress, especially in the context of the tumor microenvironment (TME), can also promote dysfunction in T_ex_ cells^6^. Rapidly dividing cancer cells outgrow their vasculature, leading to poorly oxygenated regions within the tumor^7^, which can promote T cell exhaustion^8,9^. Cancer cells are highly metabolically active and outcompete T cells for essential metabolites^10^, while also secreting metabolic byproducts into the extracellular space. One of the most abundant metabolites in the TME is lactic acid, which cancer and stromal cells export as an end product of aerobic glycolysis^11^. This leads to a reduction in extracellular pH, which inhibits T cell function^12,13^. As T cell function and differentiation are intrinsically linked to various metabolic pathways, many approaches target pathways to modulate the metabolic milieu of the TME and promote antitumor immunity^14^. It remains important to understand metabolic drivers of dysfunction in tumor-infiltrating CD8^+^ T_ex_ cells to propel development of cancer treatments.

While the abundance of a metabolite in the extracellular environment is important, cells require transporters to exchange most metabolites through the plasma membrane. Metabolite transporters act as gatekeepers of metabolism, giving cells access to exchange metabolites with their environment. Members of the solute carrier (SLC) superfamily play critical roles in transporting a host of different metabolites, either into—or out of—an organelle or cell^15^. SLCs are critical in shaping the metabolic landscape of the TME, as cancer cells use these transporters to secrete metabolic end products that can suppress antitumor immunity^16^. Expression of SLCs in cancer cells enables them to outcompete immune cells for essential metabolites^17^. Which SLCs are expressed in subsets of tumor-infiltrating CD8^+^ T cells, how these SLCs affect their metabolism and whether their expression can influence differentiation into exhaustion remains unstudied.

Here, we show that tumor-infiltrating T_ex_ cells highly upregulate the *Slc16* monocarboxylate transporter (MCT) *Slc16a11* (MCT11), enabling increased uptake of monocarboxylates (such as lactic acid) upon tumor infiltration. While expression of MCT11 could be upregulated by chronic TCR stimulus, exposure to hypoxia drove greater and sustained MCT11 expression, dependent on Hif1α. Deletion of MCT11 in endogenous T cells resulted in improved effector functions within T_ex_ cells and synergy with αPD1 therapy. Given the effects of conditional knockout of MCT11 in T cells, we treated tumor-bearing mice with MCT11-blocking monoclonal antibodies and found that the blockade of MCT11 could lead to clearances as a monotherapy but could also synergize with αPD1. Thus, this study suggests SLCs can be targeted on immune cells for therapeutic benefit.

## Results

### T_ex_ cells express MCT11, promoting lactic acid metabolism

**Fig. 1:**
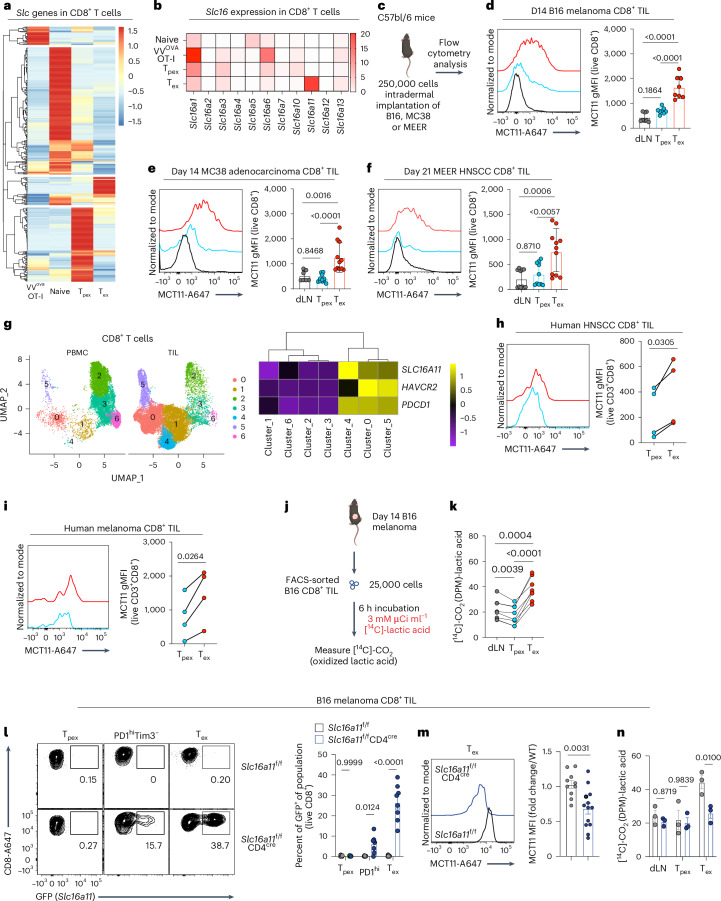
MCT11 expression enables lactic acid metabolism in T_ex_ cells. **a**, SLC superfamily gene expression from bulk RNA-seq in splenic OT-I T cells responding to vaccinia^OVA^, tumor dLN naive CD8^+^ T cells and B16-infiltrating T_pex_ (PD1^int^Tim3^-^Slamf6^hi^TOX^int^) and T_ex_ (PD1^hi^Tim3^+^Slamf6^lo^TOX^hi^) CD8^+^ T cells. **b**, Slc16 family gene transcripts per million in CD8^+^ T cell groups. **c**, A total of 250,000 B16, MC38 or MEER cells were intradermally implanted on C57BL/6 mice. **d**–**f**, A representative histogram and geometric mean fluorescence intensity (gMFI) quantification of MCT11 surface expression from ex vivo CD8^+^ T cells collected from day 14. **d**–**f**, B16 melanoma (*n* = 8) (**d**), MC38 colorectal cancer (*n* = 10) (**e**) and day 21 MEER HNSCC-bearing mice (*n* = 11) (**f**). **g**, Slc16a11 expression from single-cell sequencing in CD8^+^ T cell populations in human PBMC- and HNSCC-infiltrating CD8^+^ T cells^22,23^. **h**,**i**, Representative histogram and quantification of MCT11 expression in CD8^+^ TIL from human HNSCC (*n* = 4) (**h**) and melanoma (*n* = 4) (**i**). **j**, Experimental outline of [^14^C]-lactic acid oxidation, where 25,000 B16 dLN CD8^+^ T cells and tumor-infiltrating T_pex_ and T_ex_ cells were sorted by fluorescence activated cell sorting (FACS) and cultured for 6 h in [^14^C]-lactic acid to measure the amount converted to [^14^C]-CO_2_. **k**, [^14^C]-lactic acid oxidation converted to [^14^C]-CO_2_ in disintegrations per minute (dpm) in dLN CD8^+^ T cells and tumor-infiltrating T_pex_ and T_ex_ cells (*n* = 7). *Slc16a11*^f/f^*CD4*^cre^ (*n* = 8) and *Slc16a11*^f/f^ littermate controls (*n* = 8) were implanted with B16 and sacrificed on day 14. **l**, Representative flow cytometry plots and quantification of MCT11 (via GFP expression) in T_pex_, PD1^hi^ and T_ex_ T cell populations in *Slc16a11*^f/f^*CD4*^cre^ (*n* = 8) and *Slc16a11*^f/f^ mice (*n* = 8). **m**, A representative histogram and mean fluorescence intensity (MFI) quantification of MCT11 in T_ex_ cells from *Slc16a11*^f/f^*CD4*^cre^ (*n* = 13) and *Slc16a11*^f/f^ mice (*n* = 10). **n**, [^14^C]-lactic acid oxidation in dLN CD8^+^ T cells and B16-infiltrating T_pex_ and T_ex_ cells from *Slc16a11*^f/f^*CD4*^cre^ (*n* = 3) and *Slc16a11*^f/f^ (*n* = 3) mice. The data represent three independent experiments for **d**–**f**, **l** and **n** and four for **h**, **i**, **k** and **m**. The error bars indicate the mean ± the standard error of the mean. The statistical analysis was performed by a one-way ANOVA with Tukey’s multiple comparisons test for **d**–**f** and **k**, paired two-tailed Student’s *t*-tests for **h** and **i**, two-way ANOVA with Šidák’s multiple comparison for **l** and **n** or unpaired two-tailed Student’s *t*-tests for **m**. Panels **c** and **j** were created with BioRender.com. Source data

**Extended Data Fig. 1:**
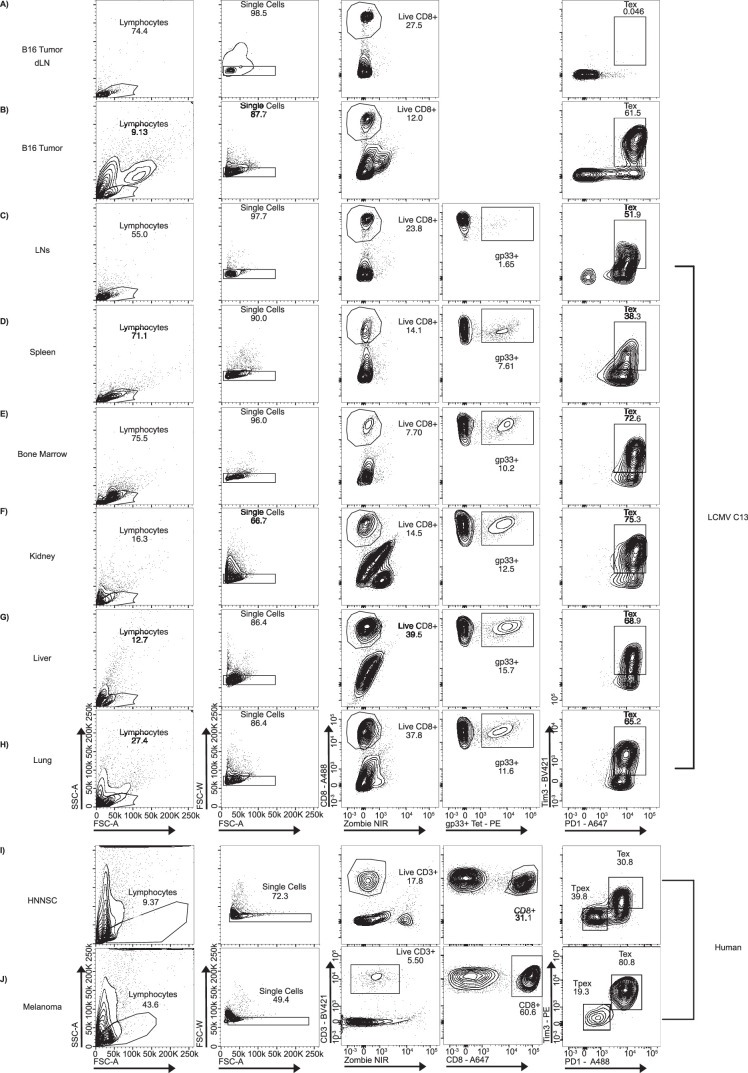
Gating strategy in mouse model and human samples. Gating strategy from total sample to live CD8^+^PD1^+^Tim3^+^ T cells from **a)** dLN and **b)** B16 tumors. Gating strategy from total sample to live CD8^+^gp33tet^+^PD1^+^Tim3^+^ T cells from murine LCMV C13 infected **c)** LNs, **d)** spleen, **e)** bone marrow, **f)** kidney, **g)** liver and **h)** lung. Gating strategy from total sample to live CD8^+^ T cells in **i)** human HNNSC and **j)** melanoma.

**Extended Data Fig. 2:**
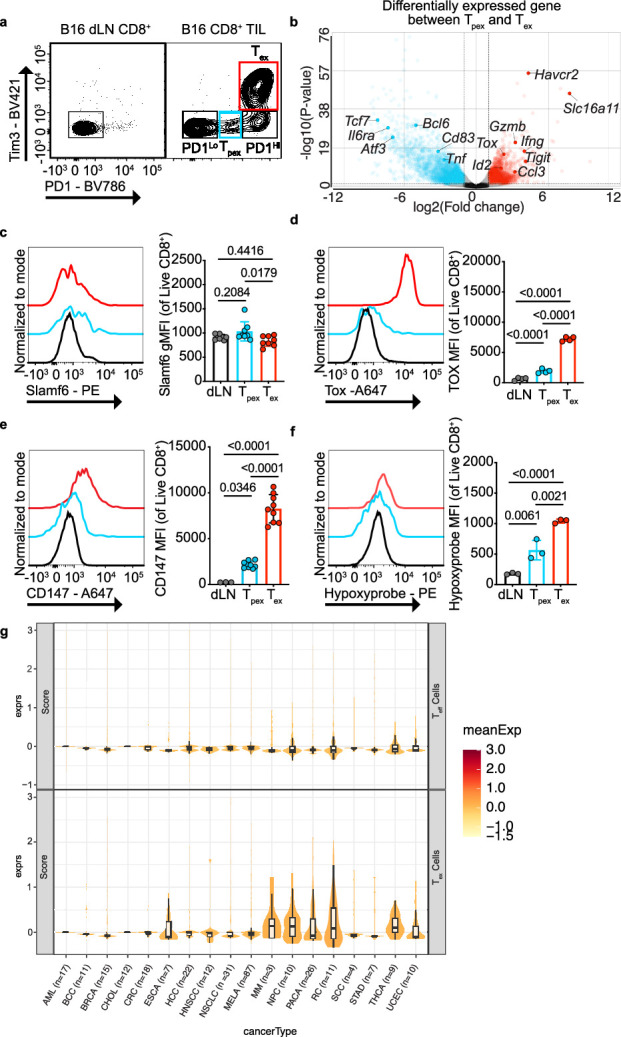
Terminally exhausted T cells differentially express solute carriers. **a)** Representative flow cytometry plot of PD1 and Tim3 expression on CD8^+^ T cells in day 14 B16 tumors and dLNs. **b)** Volcano plot of differentially expressed genes in progenitor vs terminally exhausted T cells. **c)** Representative histogram and quantification of Slamf6 expression in B16 dLN CD8^+^ T cells, and B16 tumor infiltrating T_pex_ and T_ex_ (n = 8 for all groups). **d)** Representative histogram and quantification of Tox expression in B16 dLN CD8^+^ T cells (n = 4), and B16 tumor infiltrating T_pex_ (n = 4) and T_ex_ cells (n = 4). **e)** Representative histogram and quantification of CD147 expression in dLN CD8^+^ (n = 3), and B16 tumor infiltrating T_pex_ (n = 9) and T_ex_ cells (n = 9). **f)** Representative histogram and quantification of hypoxyprobe staining in B16 dLN CD8^+^ T cells (n = 3), and B16 tumor infiltrating T_pex_ (n = 3) and T_ex_ cells (n = 3). **g)** Expression of *SLC16A11* from single cell analysis in human effector and T_ex_ cells in various human tumors. Data represent one (**d**,**f**) or two (**c**,**e**) independent experiments. Error bars indicate +/− standard error of mean (SEM). Statistical analysis performed by one way analysis of variance (ANOVA) with Tukey’s multiple comparisons test (**c**-**f**). Source data

T_ex_ cells exist in a state of metabolic dysfunction in the TME, due to disrupted mitochondrial biogenesis^18^, hypoxia^19^ and competition with tumor cells for essential metabolites^10,17^. As cells take up a substantial proportion of their metabolites using members of the SLC superfamily^20^, we sought to determine which SLCs were expressed in tumor-infiltrating T_ex_ cells. We compared the transcriptome of splenic OT-I (transgenic T cell with TCR specific to ovalbumin (OVA)) effector T cells responding to vaccinia^OVA^, tumor-draining lymph node (dLNs) naive CD8^+^ T cells, T_pex_ cells (CD8^+^PD1^int^Tim3^−^Slamf6^hi^TOX^int^) and T_ex_ cells (CD8^+^PD1^hi^Tim3^+^Slamf6^lo^TOX^hi^) from murine B16 melanoma tumors (Extended Data Figs. 1 and 2a–d)^21^. SLCs were differentially expressed during distinct stages of activation, highlighting that various subsets are engaged in distinct metabolic processes requiring a unique set of transporters (Fig. 1a). Consistent with the notion that T cells compete poorly for nutrients in the TME, T_ex_ cells repressed the majority of SLCs (Fig. 1a). However, a subset of SLCs were preferentially expressed in T_ex_ cells. One of the most differentially expressed SLCs in T_ex_ cells is a member of the *Slc16* MCT family, *Slc16a11* (MCT11), the dominant MCT expressed in T_ex_ cells (Fig. 1b). In fact, *Slc16a11* was one of the most differentially expressed genes when comparing T_ex_ with T_pex_ cells and was enriched more than canonical exhaustion genes such as *Tox*, *Ifng* and *Gzmb* (Extended Data Fig. 2b). Flow cytometric analysis using a mouse and/or human cross-reactive antibody on CD8^+^ tumor infiltrating lymphocytes (TIL) from B16, MC38 and MEER (a model of human papillomavirus-positive head and neck squamous cell carcinoma (HNSCC)) revealed MCT11 to be highly expressed on the surface of T_ex_ cells (Fig. 1c–f). Analysis of previously published^22,23^ single-cell sequencing data from human CD8^+^ intratumoral T cells showed that human T_ex_ cells also expressed *SLC16A11*, with a particularly high expression in HNSCC-infiltrating CD8^+^ T cells coexpressing *HAVCR2* and *PDCD1*, with minimal representation of these clusters in peripheral blood mononuclear cells (PBMCs) (Fig. 1g). We confirmed the expression of MCT11 protein on the surface of human T_ex_ cells from HNSCC and melanoma via flow cytometry (Fig. 1h,i). Using previously published single-cell sequencing data^24^, we were able to identify *SLC16A11* upregulation in T_ex_ cells from various human cancers. *SLC16A11* expression was upregulated in T_ex_ cells from pancreatic cancer, multiple myeloma, ovarian cancer and renal cancer (Extended Data Fig. 2g)^24^. These data highlight MCT11 as a novel surface protein on tumor-infiltrating T_ex_ cells.

**Extended Data Fig. 3:**
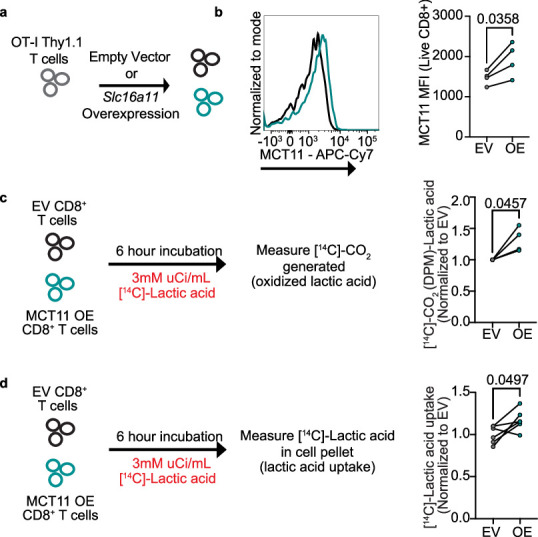
MCT11 overexpression increases lactic acid metabolism in CD8 + T cells. **a)** OT-I T cells were retrovirally transduced with an MCT11 overexpression vector. **b)** Representative histogram and quantification of MCT11 expression in OT-I T cells transduced with EV control plasmid (n = 4) or an MCT11 overexpression plasmid (n = 4). **c)** Experimental outline and quantification of [^14^C]-Lactic acid oxidation experiment in EV (n = 4) or MCT11 OE T cells (n = 4). **d)** Experimental outline and quantification of [^14^C]-Lactic acid uptake experiment in EV (n = 6) or MCT11 OE T cells (n = 6). Data represent four independent experiments. Error bars indicate +/− SEM. Statistical analysis performed by paired two-tailed Student’s T tests (**b**-**d**). Source data

MCT11 is a member of the *Slc16* family, which are MCTs that mediate transport of metabolites such as pyruvate, lactate and ketone bodies^25^. MCT11 was first described in 2014; polymorphisms in the gene led to susceptibility to type 2 diabetes^26^. Through various follow up studies^27,28^, it has been shown that MCT11 is a proton-coupled type-1 MCT that is chaperoned to the cell surface by basigin, also known as CD147 (ref. ^27^), and highly expressed in T_ex_ cells (Extended Data Fig. 2e)^29^. We have previously shown regulatory T cells in the TME take up and metabolize lactic acid via *Slc16a1* (MCT1)^30^, so we investigated whether T_ex_ cells could take up lactic acid through MCT11, as lactic acid is present in high concentrations in the TME^11,31^. We first overexpressed MCT11 in wild-type (WT) CD8^+^ T cells and pulsed these cells with [^14^C]-lactate to measure its uptake and oxidation to [^14^C]-CO_2_ (Extended Data Fig. 3a,b). [^14^C]-lactate uptake and oxidation to CO_2_ was increased in MCT11 overexpressing cells over empty vector (EV)-expressing controls (Extended Data Fig. 3c,d), indicating lactate was being taken and metabolized through the tricarboxylic acid (TCA) cycle in MCT11-expressing cells. Next, we asked whether there was a difference in ability to metabolize lactic acid in CD8^+^ TIL populations. We sorted T_ex_ and T_pex_ cells from B16 melanoma tumors, as well as dLN CD8^+^ T cells and pulsed them with [^14^C]-lactate for 6 h (Fig. 1j). We found T_ex_ cells, which express MCT11, had increased [^14^C]-lactate oxidation to [^14^C]-CO_2_, compared with T_pex_ cells, which do not express it (Fig. 1k).

**Extended Data Fig. 4:**
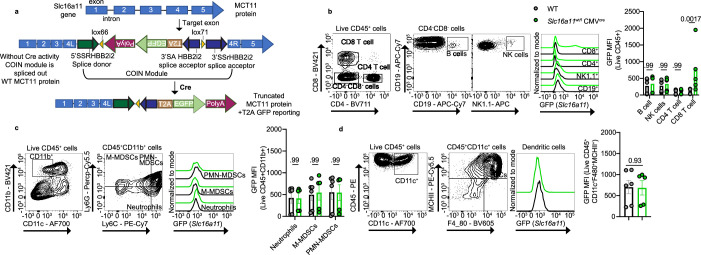
MCT11 expression is confined to exhausted T cells among tumor infiltrating immune populations. **a)** Cartoon of *Slc16a11* exons in WT and *Slc16a11*^f/f^ mice where Cre expression leads to the transcription of a truncated MCT11 protein with simultaneous GFP expression. **b)** Representative flow cytometry plot, histogram and quantification of MCT11 (via GFP reporter expression) in CD8, CD4, NK and B cells infiltrating B16 tumors on WT (n = 6) and *Slc16a11*^wt/f^CMV^cre^ (n = 5) mice. **c)** Representative flow cytometry plot, histogram and quantification of MCT11 (via GFP reporter expression) in B16 infiltrating monocytes on WT (n = 6) and *Slc16a11*^wt/f^CMV^cre^ (n = 5) mice. **d)** Representative flow cytometry plot, histogram and quantification of MCT11 (via GFP reporter expression) in B16 infiltrating dendritic cells on WT (n = 6) and *Slc16a11*^wt/f^CMV^cre^ (n = 5) mice. Data represent two independent experiments. Error bars indicate +/− SEM. Statistical analysis performed by two-way ANOVA with Sidak’s multiple comparisons test (**b**-**c**) or by unpaired two-tailed Student’s T tests (**d**). Source data

To further understand the role of MCT11 in T_ex_ cell biology, we generated a conditional knockout by inversion (COIN)^32^ *Slc16a11*^f/f^ mouse. This system works by targeting a gene of interest and interrupting its transcription by inverting a COIN module into an exon of the gene. We did this by inserting an artificial intron with a T2A–eGFP COIN module into the fourth exon of the *Slc16a11* antisense strand, flanked by lox71 and lox66 and splicing sequences. Without Cre activity, the COIN module is spliced out, generating WT *Slc16a11* mRNA and protein. Under Cre activity, lox66 and lox71 flanking enables the inversion of the T2A–eGFP COIN module into the fourth exon of Slc16a11 (ref. ^33^), generating a truncated nonfunctional MCT11 protein and reporting of this event via green fluorescent protein (GFP) expression (Extended Data Fig. 4a). We, thus, generated a system where we could knockout the function of MCT11 and identify would-be MCT11-expressing cells by GFP fluorescence.

**Extended Data Fig. 5:**
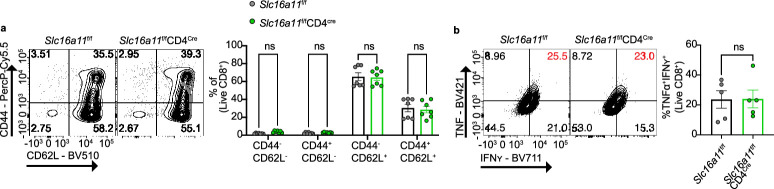
CD8^+^ T cells from *Slc16a11*^f/f^*CD4*^Cre^ mice have no developmental or functional differences until they become exhausted. **a)** Representative flow cytometry plot and quantification of CD44 and CD62L populations in CD8^+^ T cells isolated from LNs of 10-week-old *Slc16a11*^f/f^ (n = 7) and *Slc16a11*^f/f^*CD4*^Cre^ mice (n = 7) **b**) Representative cytogram and quantification of TNF and IFNʏ populations in CD8 + T cells isolated from LNs of *Slc16a11*^f/f^*CD4*^Cre^ (n = 5) mice and littermate controls (n = 5). Data represent two independent experiments. Error bars indicate +/− SEM. Statistical analysis performed by two -way ANOVA with Sidak’s multiple comparisons (**a**) or by unpaired two-tailed Student’s T tests (**b**). Source data

First, we generated a *Slc16a11*^wt/f^CMV^cre^ line, which carry one functional allele of the gene, and searched for MCT11 expression via GFP fluorescence in tumor-infiltrating immune cells. Analysis of day 14 B16-infiltrating immune cells from *Slc16a11*^wt/f^CMV^cre^ mice confirmed that CD8^+^ T cells expressed MCT11. Further, MCT11 was not expressed by other tumor-infiltrating immune cells, as tumor-infiltrating CD4^+^ T cells, B cells, natural killer cells, dendritic cells, polymorphonuclear-myleoid derived suppressor cells (PMN-MDSCs), monocytic-myeloid derived suppressor cells (M-MDSCs) and neutrophils (Extended Data Fig. 4). *Slc16a11*^f/f^ mice were crossed with *CD4*^*cre*^ mice to generate a T cell-conditional knockout. As expected, because *Slc16a11* is not expressed until T cells reach terminal exhaustion, there were no differences in development and function directly ex vivo (Extended Data Fig. 5a,b). Analysis of GFP expression in CD8^+^ TIL subsets from B16-bearing *Slc16a11*^f/f^ and *Slc16a11*^f/f^CD4^cre^ mice confirmed our previous findings that MCT11 was expressed in T_ex_ cells (~25% of cells) but not T_pex_ cells (Fig. 1l). In addition, this analysis revealed that MCT11 expression begins in PD1^hi^Tim3^−^ cells, during the transition from progenitor to terminal exhaustion (Fig. 1l). Antibody staining confirmed knockout of MCT11 in T_ex_ cells from *Slc16a11*^f/f^CD4^cre^ (Fig. 1m). We then asked whether T_ex_ cells lacking MCT11 have a reduced ability to oxidize lactic acid. Indeed, conditional deletion of MCT11 led to decreased lactic acid oxidation in T_ex_ cells, reducing it essentially to the level of T_pex_ cells (Fig. 1n). Thus, T_ex_ cells uniquely upregulate MCT11, which enables the flux and metabolism of monocarboxylates, such as lactic acid.

### MCT11 expression is driven by chronic TCR stimulus

**Fig. 2:**
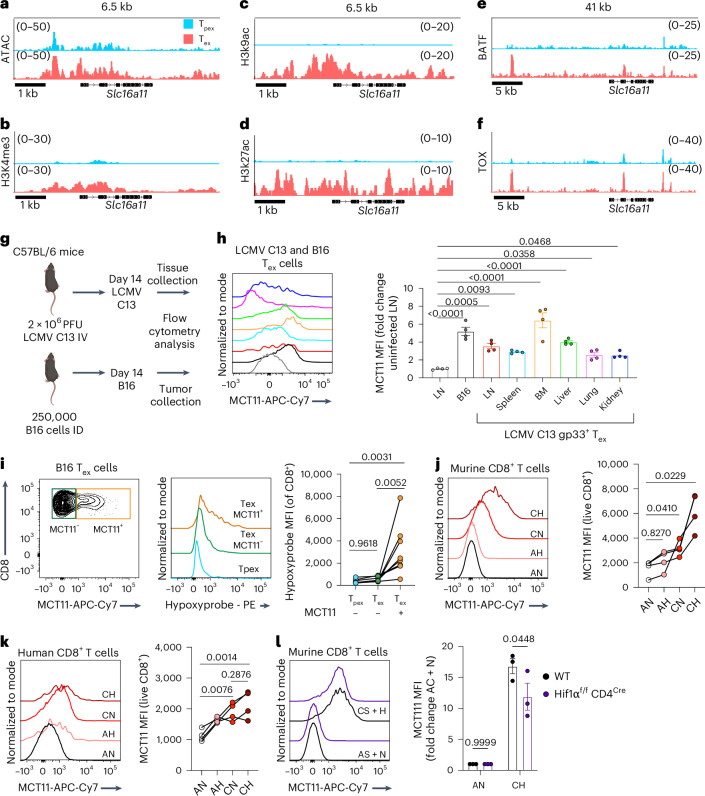
MCT11 expression is driven by continuous TCR stimulation. **a**, ATAC sequencing of the *Slc16a11* locus in T_pex_ and T_ex_ cells responding to B16-F10 (ref. ^4^). **b**–**f**, CUT&RUN of H3K4me3 (**b**), H3K9ac (**c**), H3K27ac (**d**), BATF (**e**) and Tox (**f**) at the *Slc16a11* locus in B16 melanoma-infiltrating T_pex_ and T_ex_ cells^21^. **g**, C57BL/6 mice were intravenously (IV) infected with 2 × 10^6^ PFU of LCMV C13 or intradermally (ID) implanted with 250,000 B16 melanoma cells. A total of 14 days later, LN, spleen, BM, liver, lung and kidney were collected from LCMV C13-infected mice, and the tumors were collected from B16 melanoma-bearing mice. **h**, A representative histogram and quantification of MCT11 surface expression from ex vivo T_ex_ cells isolated from B16 melanoma-bearing mice (*n* = 4) or tissues from LCMV C13-infected mice (*n* = 4). **i**, Hypoxia experienced by D14 B16 melanoma-infiltrating CD8^+^ T cells expressing surface MCT11 (*n* = 7). **j**, A representative histogram and quantification of MCT11 surface expression in murine CD8^+^ T cells cultured under acute stim (A, 1:1 stimulatory beads to T cell ratio for 24 h) and normoxia (N, 20% O_2_), A and hypoxia (H, 1.5% O_2_), continuous stim (C, 10:1 stimulatory beads to T cell ratio for 6 days) and N, or C and H (*n* = 4). **k**, A representative histogram and quantification of MCT11 surface expression of human CD8^+^ T cells cultured under the previously described conditions (*n* = 4). **l**, A representative histogram and quantification of MCT11 expression on CD8^+^ from WT (*n* = 3) and Hif1α^f/f^CD4^Cre^ (*n* = 3) mice cultured in C + H. The data represent two independent experiments for **h** and three for **i**–**l**. The error bars indicate the mean ± the standard error of the mean. The statistical analysis was performed by a repeated measures one-way ANOVA with Dunnet’s multiple comparison for **h**, one-way ANOVA with Tukey’s multiple comparison for **i**–**k** or two-way ANOVA with Šidák’s multiple comparison for **l**. MFI, mean fluorescence intensity. Panel **g** was created with BioRender.com. Source data

**Extended Data Fig. 6:**
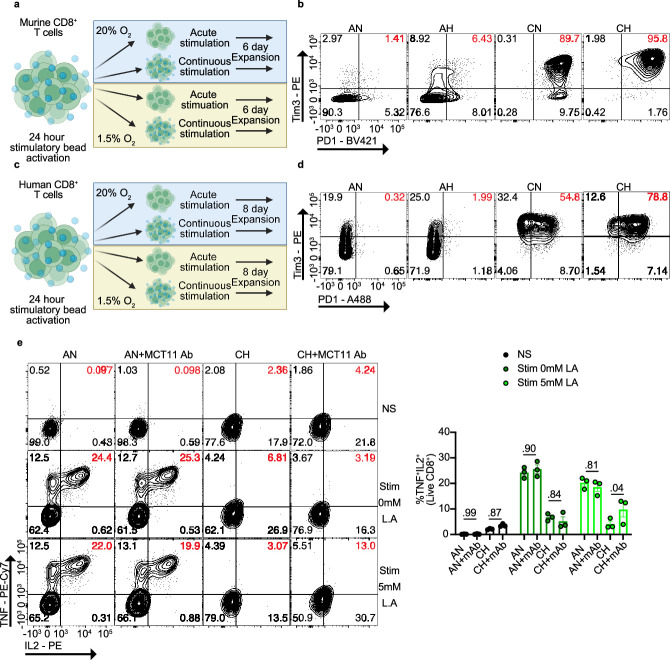
Continuous stimulation under hypoxia drives an exhaustion-like state. **a)** Murine CD8^+^ T cells were activated with CD3/CD28 stimulatory beads at a 1:1 ratio for 24 hours, and then expanded for 6 days w/out beads or at a 10:1 (B:T) ratio in 20% O_2_ or %1.5 O_2_. **b)** Flow cytometry plot of PD1 and Tim3 expression in murine CD8^+^ T cells after 6 days in culture under acute stimulation (A) and normoxia (N), A and hypoxia (H),continuous stimulation (C) and N, or C and H. **c)** Human CD8 + T cells were activated with CD3/CD28 stimulatory beads at a 1:1 ratio for 24 hours, and then expanded for 8 days without beads or at a 10:1 (B:T) ratio in 20% O2 or %1.5 O2. **d)** Flow cytometry plots of PD1 and Tim3 expression in human CD8^+^ T cells after 8 days in culture in the described conditions. **e)** Representative flow cytometry plot and tabulation of the percent of cells producing TNF and IL2, when stimulated for 5 hours with αCD3 (3 µg/mL) and αCD28 (2 µg/mL) in the presence of 5 mM lactic acid +/− MCT11 antibody (40 µg/mL) (n = 3 for all samples). Data represent one experiment. Error bars indicate +/− SEM. Statistical analysis performed by two-way ANOVA with Tukey’s multiple comparison (**e**). (**a**) Created in BioRender. Peralta, R. (2023) BioRender.com/r15f775. Source data

Given the distinct expression of MCT11 in T_ex_ cells in tumors, we next wanted to determine what drove *Slc16a11* expression by examining the epigenetic landscape of its locus. An analysis of previously published^4^ ATAC sequencing revealed *Slc16a11* was more accessible in T_ex_ than T_pex_ cells from B16 tumors (Fig. 2a). Further, analysis of our previously published^21^ CUT&RUN data showed that the locus of *Slc16a11* in T_ex_ cells harbors the permissive histone modifications H3K4me3, H3K9ac and H3K27ac but not in T_pex_ cells (Fig. 2b–d). In addition, the *Slc16a11* locus is active and bound by the exhaustion-associated transcription factors BATF and TOX in T_ex_ cells (Fig. 2e,f). We next asked whether MCT11 expression was exclusive to tumor-infiltrating T_ex_ cells. Lymphocytic choriomeningitis virus clone 13 (LCMV C13) is a chronic viral infection that has been widely used to study T cell exhaustion. Given that LCMV C13 is a systemic infection, we searched for MCT11 expression in T_ex_ cells from different tissues of LCMV C13-infected mice and compared it with B16-infiltrating T_ex_ cells (Fig. 2g). We found that MCT11 was expressed in T_ex_ cells from the lymph nodes (LNs), spleen, kidney, liver, lung and bone marrow (BM) of LCMV C13-infected mice at varying levels of expression in the different tissues (Fig. 2h) These data highlight MCT11 expression as part of the T cell exhaustion program and suggested there may be tissue specific factors further driving the expression of MCT11 in T_ex_ cells. The highest expression of MCT11 was in LCMV C13 T_ex_ cells from the BM, a tissue with hypoxic niches, such as tumors^34,35^. Our lab and others have shown that T_ex_ cells experience higher levels of hypoxia in the TME than T_pex_ cells (Extended Data Fig. 2f)^8,19,36,37^. Therefore, we investigated the levels of hypoxia experienced by MCT11^+^ T_ex_ cells by injecting B16-bearing mice with pimonidazole, a hypoxia tracer^38^. We found MCT11^+^ T_ex_ cells had greater hypoxia exposure than MCT11^−^ T_ex_ cells (Fig. 2i), suggesting hypoxia could be a tissue specific factor that promotes MCT11 expression. To explore this in a reductionist fashion, we cultured CD8^+^ T cells under continuous TCR stimulus (protocol previously published), sufficient to drive an exhausted-like state (Extended Data Fig. 6), which drove MCT11 expression in murine and human CD8^+^ T cells (Fig. 2j,k). MCT11 expression was increased further when T cells receiving continuous TCR stimulus were cultured under hypoxia (Fig. 2j,k). Given the increased expression of MCT11 under hypoxia, we hypothesized that Hif1α may promote expression of MCT11 in T_ex_ cells. While CD8^+^ T cells from *Hif1α*^f/f^CD4^cre^ upregulated MCT11 when cultured under chronic TCR stimulus and hypoxia, there was a significant decrease in MCT11 expression when compared with WT CD8^+^ T cells (Fig. 2l), consistent with previous findings that *Hif1α* is not solely responsible for hypoxia-induced potentiation of T cell exhaustion. Altogether, our data suggest that the core exhaustion program primes the *Slc16a11* locus for expression, and exposure to tissue factors such as hypoxia, through *Hif1α*, further promotes MCT11 expression.

### MCT11 enforces dysfunction in T_ex_ cells

**Extended Data Fig. 7:**
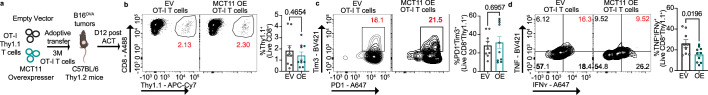
MCT11 overexpression accelerates T cell dysfunction in the TME. **a)** OT-I Thy1.1 T cells were retrovirally transduced with an MCT11 overexpression vector (OE) or control empty vector (EV), and 3 million T cells were adoptively transferred into day 7 B16^OVA^ bearing C57BL/6 Thy1.2 mice. **b)** Representative flow plot and quantification of percentage of CD8+Thy1.1^+^ transduced with EV (n = 9) or OE (n = 10) (transferred OT-Is) infiltrating B16^OVA^ tumors. **c)** Representative flow plot and quantification of PD1^+^Tim3^+^ population in adoptively transferred EV (n = 9) or OE (n = 10) OT-I T cells. **d)** Representative flow plot and quantification of TNF^+^IFNʏ^+^ population in adoptively transferred EV (n = 9) or OE (n = 10) T cells. Data represent three independent experiments. Error bars indicate +/− SEM. Statistical analysis performed by unpaired two-tailed Student’s T tests (**b**-**d**). (**a**) Created in BioRender. Peralta, R. (2024) BioRender.com/k52v484. Source data

We next sought to determine the contribution of MCT11-mediated metabolite uptake in the function and fate of T_ex_ cells. First, we retrovirally overexpressed MCT11 in OT-I (Thy1.1) T cells before adoptive transfer into B16^OVA^-bearing mice (Thy1.2) (Extended Data Fig. 7a). Overexpression of MCT11 resulted in accelerated functional exhaustion, as these T cells had decreased TNF and interferon γ (IFNγ) production, although no appreciable changes were observed in tumor infiltration or coinhibitory marker expression when compared with EV controls (Extended Data Fig. 7b–d). These data suggested that MCT11, and by extension lactate uptake, could be driving dysfunction within T_ex_ cells.

**Fig. 3:**
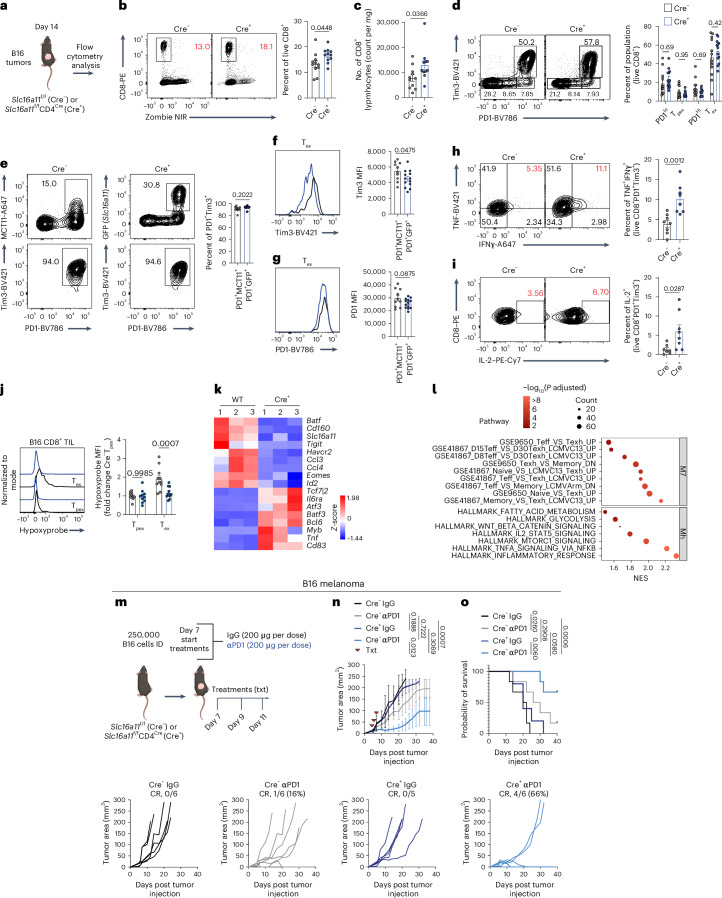
MCT11 enforces dysfunction in T_ex_ cells. **a**, *Slc16a11*^f/f^*CD4*^cre^ (Cre^+^) and littermate *Slc16a11*^f/f^ (Cre^−^) controls were injected with 250,000 B16 melanoma and sacrificed on day 14 for tumor collection and flow cytometry analysis. **b**,**c**, Representative flow cytometry plots and quantification of percentage (**b**) and total counts of live CD8^+^ T cells infiltrating B16 tumors (**c**) in Cre^−^ (*n* = 11) and Cre^+^ mice (*n* = 11). **d**, A representative flow cytometry plot and quantification of PD1 and Tim3 populations in ex vivo B16 melanoma-infiltrating CD8^+^ T cells in Cre^−^ (*n* = 12) and Cre^+^ mice (*n* = 13). **e**, A representative flow cytometry plot and quantification of T_ex_ cells in PD1^+^MCT11^+^ and PD1^+^GFP^+^ T cells from Cre^−^ (*n* = 10) and Cre^+^ (*n* = 13) mice, respectively. **f**,**g**, Representative histograms and quantification of Tim3 (**f**) and PD1 MFI (**g**) in PD1^+^MCT11^+^ and PD1^+^GFP^+^ T cells from Cre^−^ (*n* = 10) and Cre^+^ (*n* = 13) mice, respectively. **h**,**i**, A representative flow cytometry plot and quantification of TNF and IFNʏ (**h**) and IL-2 (**i**) in B16-infiltrating T_ex_ cells from Cre^−^ (*n* = 8) and Cre^+^ mice (*n* = 8) after 6 h of stimulation with αCD3 (3 μg ml^−1^) and αCD28 (2 μg ml^−1^). **j**, A representative cytogram and quantification of hypoxyprobe in T_pex_ and T_ex_ cells from Cre^−^ and Cre^+^ mice. **k**, Heat map of differentially expressed genes between T_ex_ cells from B16 tumors on Cre^+^ and WT mice. **l**, A gene set enrichment analysis of selected immunologic signature and hallmark gene sets in Cre^+^ T_ex_ cells over WT T_ex_ cells. A multiple comparisons correction was performed using the Benjamini–Hochberg method. **m**, Cre^+^ and Cre^−^ mice were intradermally (ID) implanted with 100,000 tumor cells and treated (txt) on days 7, 9 and 11 with αPD1 or isotype control mAb. **n**,**o**, Tumor growth with complete responses (CR) (**n**) and survival curve (**o**) of B16 melanoma on Cre^−^ and Cre^+^ mice. The data represent three independent experiments. The error bars indicate the mean ± the standard error of the mean. The statistical analysis was performed by unpaired two-tailed Student’s *t*-tests for **b**, **c** and **e**–**i**, by a two-way ANOVA with Tukey’s multiple comparison for **d**, **j** and **n** or by a log rank Mendel–Cox test for **o**. Panels **a** and **m** were created with BioRender.com. Source data

To further interrogate the role of MCT11 in T cell exhaustion, we inoculated *Slc16a11*^f/f^ and *Slc16a11*^f/f^CD4^cre^ mice with B16 melanoma and studied the infiltrating TIL via flow cytometry (Fig. 3a). Tumors from *Slc16a11*^f/f^CD4^cre^ mice had a significant increase in infiltrating CD8^+^ T cells by percentage and total counts (Fig. 3b,c). MCT11 deficiency in CD8^+^ T cells did not lead to any numerical changes in the PD1^lo^, T_pex_, PD1^hi^ and T_ex_ populations (Fig. 3d), suggesting T cells still progressed to an exhausted surface phenotype. However, as not all T_ex_ cells express MCT11, we compared the exhaustion profile of GFP^+^ T_ex_ cell population from *Slc16a11*^f/f^CD4^cre^ mice to MCT11^+^ T_ex_ cells from *Slc16a11*^f/f^ controls. While we found no difference in the percentage of the T_ex_ population (Fig. 3e), we found T_ex_ cells lacking MCT11 (GFP^+^ cells) had a significant decrease in the per-cell expression of Tim3 (Fig. 3f) and a similar trend in PD1 expression (Fig. 3g). Further, we found improved T cell functionality upon MCT11 deletion, as T_ex_ cells from *Slc16a11*^*f*/f^CD4^cre^ mice displayed increased polyfunctionality, by production of TNF and IFNγ, as well as increased production of IL-2 upon restimulation with αCD3/αCD28 (Fig. 3h,i). MCT11’s substrate, lactic acid, is most highly enriched in hypoxic tumor niches. Thus, we asked whether MCT11 facilitated persistence of T_ex_ cells in hypoxic niches of the tumor. Infusion of mice with pimonidazole revealed that T_ex_ cells from *Slc16a11*^f/f^CD4^cre^ experienced less hypoxia than T_ex_ cells from littermate controls, while no difference was observed in T_pex_ (Fig. 3j). These data suggest MCT11-mediated lactate uptake may support T_ex_ cell accumulation in hypoxic regions, such that its deletion results in T_ex_ cells residing in more oxygenated regions where their effector functions are improved.

RNA sequencing (RNA-seq) on MCT11-deficient T_ex_ cells revealed increased expression of genes associated with T_pex_ cells (for example, *Myb*, *Cd83* and *Bcl6*) and decreased expression of genes associated with the terminal exhausted fate (for example, *Eomes*, *Havcr2*, *Id2 and Ccl3*) (Fig. 3k). Performing a pathway enrichment analysis comparing *Slc16a11*^f/f^CD4^cre^ to WT T_ex_ cells confirmed that T_ex_ cells lacking functional MCT11 harbored a transcriptome enriched for T_pex_ genes (Fig. 3l). These data suggested that T_ex_ cells from *Slc16a11*^f/f^CD4^cre^ mice would be primed for a superior response to αPD1 therapy. While B16-bearing *Slc16a11*^f/f^CD4^cre^ mice treated with isotype control did not grow significantly slower in comparison with WT controls (Fig. 3m), when treated with αPD1, conditional deletion of MCT11 led to 66% (4/6) complete responders (CRs), compared with only 16% CRs (1/6) in WT controls (Fig. 3n). These data suggest a metabolite flux via MCT11 promotes effector dysfunction in exhausted tumor-infiltrating CD8^+^ T cells and that it could be targeted to promote CD8^+^ T cell-mediated antitumor immunity.

### MCT11 antibody blockade reduces tumor burden in mice

**Fig. 4:**
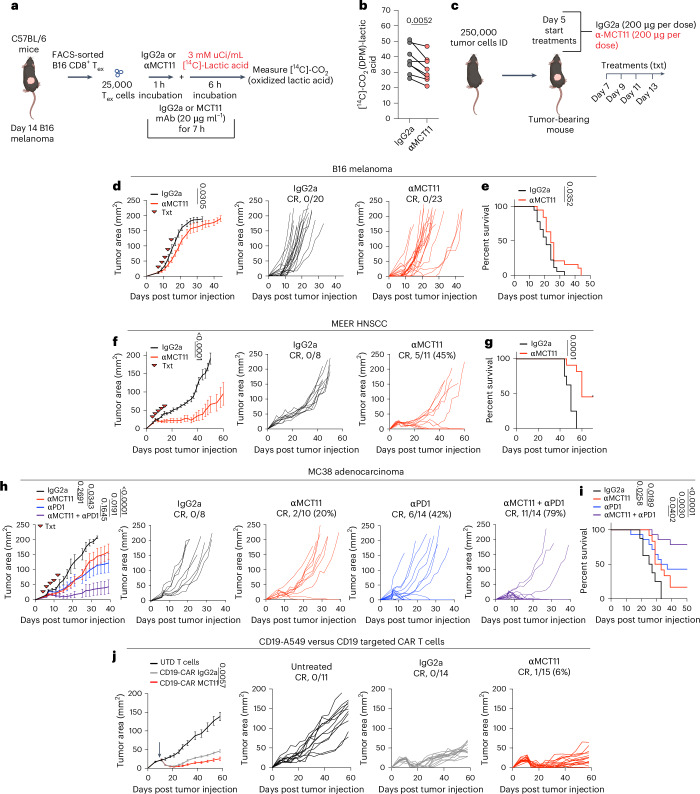
MCT11 antibody blockade reduces tumor burden in mice. **a**, T_ex_ cells were sorted from B16 melanoma tumors and cultured at 25,000 cells per well in the presence of 20 μg ml^−1^ of IgG2a or αMCT11 for 1 h before the addition of 3 mM [^14^C]-lactic acid for 6 h (assay total, 7 h). **b**, Ex vivo [^14^C]-lactic acid oxidation in tumor-infiltrating T_ex_ cells in the presence of αMCT11 or isotype control (*n* = 8). **c**, An experimental outline for tumor growth curves with tumor cell lines, where mice were injected with 250,000 tumor cells intradermally (ID) and treated (txt) with αMCT11 or isotype control (200 μg per dose, five doses total) after day 5 of tumor growth. **d**–**g**, The tumor growth curves and survival curves of B16 (**d** and **e**, respectively) and MEER-bearing C57BL/6 (**f** and **g**, respectively) mice treated with αMCT11 therapy. **h**,**i**, Tumor growth (**h**) and survival (**i**) curve of mice injected ID with 250,000 MC38 cells and treated with isotype control, αMCT11, αPD1 or combination therapy (200 μg per dose, five doses total). **j**, A tumor growth curve of CD19-A549-bearing NSG mice adoptively transferred with untransduced T cells (UDT) or CD19-targetting CAR-T cells treated with αMCT11 or isotype control (200 μg per dose, 12 doses total). The data represent two independent experiments for **j**, three for **d**–**i** or four for **b**. The error bars indicate the mean ± the standard error of the mean. The statistical analysis was performed by a paired two-tailed Student’s *t*-test for **b**, by a two-way ANOVA with Tukey’s multiple comparison for **d**, **f**, **h** and **j** or by a log rank Mendel–Cox test for **e**, **g** and **i**. Panels **a** and **c** were created with BioRender.com. Source data

Immune checkpoint blockade (ICB) therapies targeting PD1, CTLA4 and LAG3 on CD8^+^ TIL has proven to be a particularly effective modality of cancer treatment^39^. These agents act by altering signaling of T cells by preventing binding to their ligands. Given the inhibitory effect of lactic acid on T_ex_ cell function and the expression pattern on T_ex_ cells, we asked whether a monoclonal antibody against MCT11 may block lactic acid uptake in T_ex_ and promote antitumor immunity. To this end, we repeated the [^14^C]-lactate oxidation assay with sorted T_ex_ cells but in the presence of a monoclonal antibody targeting MCT11 (mIgG2a isotype) (Fig. 4a). Pretreatment with αMCT11 reduced the oxidation of [^14^C]-lactate in T_ex_ cells from B16 (Fig. 4b). As endogenous MCT11 deletion in T_ex_ cells resulted in superior T cell function (Fig. 3e,f), we treated tumor-bearing mice with αMCT11 therapeutically (Fig. 4c). αMCT11 resulted in a modest but significant decrease in tumor size in B16 melanoma-bearing mice and extension of survival without any CRs, similar to the effects of αPD1 in this model (Fig. 4d,e). However, we found striking single-agent activity in response to MCT11 blockade in MEER, where αMCT11 resulted in CRs in 45% of MEER-bearing mice (Fig. 4f,g). The MCT11 blockade required the presence of an adaptive immune response, as αMCT11 had no effect in *Rag-*deficient mice (Fig. 4h–k) and also suggested αMCT11 therapy’s effect was not due to blockade of MCT11 on B16 or MEER tumor cells themselves. As the αMCT11 mAb is a murine IgG2a isotype, one potential mechanism of therapeutic efficacy may be depletion of MCT11-expressing T_ex_ cells. We, thus, generated mIgG2a αMCT11 with a LALAPG mutation, preventing FcR binding and antibody dependent cellular cytotoxicity^40^. Treatment of MEER-bearing mice with the LALAPG mutant αMCT11 controlled tumor growth to the same extent as parental αMCT11 mAb (Fig. 4l,m), suggesting αMCT11 therapy functioned via a blockade. Rechallenging MCT11 blockade CRs after 1 month, in the absence of any additional therapy, resulted in tumor clearance, suggesting the MCT11 blockade resulted in the formation of immunologic memory (Fig. 4n,o). We also tested the effects of αMCT11 in MC38, an αPD1-responsive tumor model. We found that αMCT11 alone led to CRs in MC38, with 20% of mice treated with the antibody clearing tumors (Fig. 4p). In addition, combination therapy of αMCT11 and αPD1 nearly doubled the CR rate of αPD1 therapy alone, from 42% CR in the αPD1 alone therapy to 79% in combination (Fig. 4q).

**Extended Data Fig. 8:**
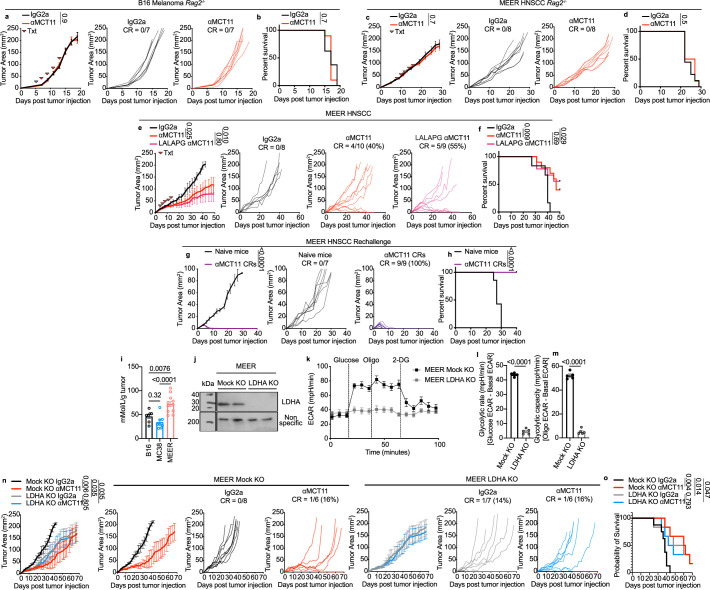
αMCT11 therapy requires T cells and is driven by lactate metabolism in cancer. Tumor growth curve and **b)** survival curve of B16 bearing *Rag2*^-/-^ mice treated with isotype control (n = 7) or αMCT11 (n = 7). **c)** Tumor growth curve and **d)** survival curve of MEER bearing *Rag2*^−/−^ mice treated with isotype control (n = 8) or αMCT11 (n = 8. **e)** Tumor growth and **f)** survival curve of MEER bearing mice treated with isotype control (n = 8), αMCT11 (n = 10) or LALAPG αMCT11 (n = 9). **g)** MEER tumor growth and **h)** survival curve of naïve mice (n = 7) and mice rechallenged 1 month after complete responses to MCT11 therapy (n = 9). **i)** Lactate concentrations in TIF of B16 (n = 7), MC38 (n = 8) and MEER (n = 11) tumors. **j)** Immunoblot of LDHA in mock KO and LDHA KO MEER tumor cells (one experiment). **k)** Extracellular acidification rate plot, **l)** glycolytic rate and **m)** glycolytic capacity in mock KO (n = 5) and LDHA KO MEER (n = 5) tumor cells. **(n)** Tumor growth and **o)** survival curves of mice injected I.D. with 250 K mock KO or LDHA KO MEER cells and treated with isotype control or αMCT11 (200 μg/dose; 5 doses total). Data represent one (**g**-**h**,**j**), two (**a**-**f**,**k**-**o**) or three (**i**) independent experiments. Error bars indicate +/− SEM. Statistical analysis performed by paired two-tailed Student’s T tests (**l**-**m**), one way ANOVA with Tukey’s multiple comparisons (**i**), Two-way ANOVA with Tukey’s multiple comparisons (**a**,**c**,**e**,**g**,**n**), or by Log Rank Mendel Cox Test (**b**,**d**,**f**,**h**,**o**). Source data

Given the differences in responses to αMCT11 therapy, we hypothesized that perhaps B16, MC38 and MEER produce metabolically distinct TMEs and, specifically, that MEER would be more lactate rich. To this end, we measured the concentration of lactic acid in the tumor interstitial fluid of B16, MC38 and MEER and found that MEER had significantly elevated levels of lactate in comparison with B16 and MC38 (Extended Data Fig. 8i). However, this did not prove that blockade of MCT11-mediated lactate metabolism provided a therapeutic benefit, as MCTs can transport other substrates, such as pyruvate, succinate, acetate, ketone bodies and other monocarboxylates^41^. To determine whether MCT11-targeted therapy was dependent on lactate metabolism, we performed a clustered regularly interspaced short palindromic repeats (CRISPR) with CRISPR-associated protein 9 (Cas9) knockout of the glycolytic gene *Ldha* in MEER (Extended Data Fig. 8j). Indeed, LDHA-deficient MEER cells were incapable of performing extracellular acidification after glucose and oligomycin treatment (Extended Data Fig. 8k–m). We then implanted mice with mock-knockout or LDHA-knockout MEER tumors and treated with either αMCT11 therapy or isotype control, revealing treatment with αMCT11 had no therapeutic benefit in mice bearing LDHA-deficient MEER (Extended Data Fig. 8n,o) These data suggest MCT11 therapy’s efficacy is dependent on the presence of a lactate-rich TME.

Further, we asked whether MCT11 blockade could provide a therapeutic benefit for human T cells. To do this, we used a humanized chimeric antigen receptor (CAR)-T cell model system of solid tumors: CD19-expressing A549 lung cancer cells and CD19-targeting (FMC63^−^BBz) CAR-T cells. We implanted NOD.Cg-*Prkdc*^*scid*^ *Il2rg*^*tm1Wjl*^/SzJ (NSG) mice with 5 × 10^6^ CD19-A549 cells and then adoptively transferred 3 × 10^6^ CD19-CAR-T cells after 10 days of tumor growth. We then treated them with αMCT11 or isotype control twice per week until the endpoint. While both CAR-T-treated groups initially induced tumor regression, mice receiving concomitant MCT11 blockade had improved long-term tumor control (Fig. 4j). Thus, MCT11 blockade can also functionally improve human T_ex_ cells.

### MCT11 blockade promotes CD8^+^ T cell antitumor immunity

We next tested the acute effects of lactic acid on T_ex_ cells during stimulation. Using our in vitro assay of continuous TCR stimulation and hypoxia, we generated T_ex_-like cells, which express MCT11 (Extended Data Fig. 6). These cells were then restimulated in media supplemented with 0 mM or 5 mM lactic acid, with or without MCT11 antibody, for 5 h. As previously described, T cells that are continuously stimulated have reduced effector cytokine production when compared with acutely activated T cells after restimulation. The addition of 5 mM lactic acid during restimulation resulted in a reduction of TNF^+^IL-2^+^ T cells. A blockade of MCT11 during restimulation with anti-MCT11 antibody led to significantly increased percentage of polyfunctional TNF^+^IL-2^+^-producing T cells (Extended Data Fig. 6e), suggesting blockade of MCT11 desensitizes T cells of the extracellular lactate capable of acutely affecting T cell responses.

**Fig. 5:**
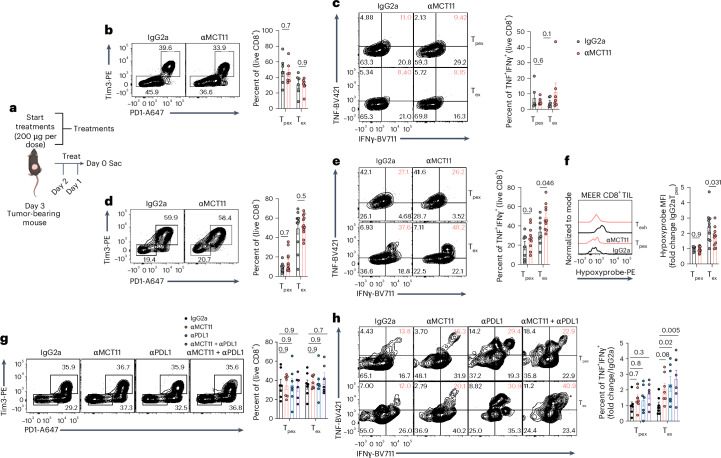
MCT11 therapy selectively enhances the polyfunctionality of T_ex_ cells. **a**, A total of 250,000 tumor cells were intradermally implanted into C57BL/6 mice and treated for three consecutive days with 200 µg per dose of αMCT11 or isotype control before sacrifice. **b**, A representative flow cytometry plot and quantification of T_pex_ and T_ex_ populations in B16 when treated with isotype control (*n* = 6) or αMCT11 (*n* = 7). **c**, A representative flow cytometry plot and quantification of TNF and IFNʏ in B16 T_pex_ and T_ex_ cells after 5 h of stimulus with αCD3 (3 µg ml^−1^) and αCD28 (2 µg ml^−1^) (isotype, *n* = 6; αMCT11, *n* = 7). **d**, A representative flow cytometry plot and quantification of PD1 and Tim3 populations staining in MEER-infiltrating T_pex_ and T_ex_ cells after antibody treatment (both groups, *n* = 11). **e**, A representative flow cytometry and quantification of TNF and IFNʏ in MEER-infiltrating T_pex_ and T_ex_ cells after 5 h of stimulus with αCD3 (3 µg ml^−1^) and αCD28 (2 µg ml^−1^) (both groups, *n* = 11). **f**, A representative histogram and quantification of hypoxyprobe in T_pex_ and T_ex_ cells from MEER tumors after antibody treatment (isotype, *n* = 10; αMCT11, *n* = 10). **g**, A representative flow cytometry plot and quantification of PD1 and Tim3 populations staining in MC38-infiltrating CD8^+^ T cells after antibody treatment (isotype, *n* = 7; αMCT11, *n* = 7; αPD1, *n* = 7; combo, *n* = 6). **h**, A representative cytogram and quantification of TNF and IFNʏ in MC38-infiltrating T_pex_ and T_ex_ cells after 5 h of stimulus with αCD3 (3 µg ml^−1^) and αCD28 (2 µg ml^−1^) (isotype, *n* = 7; αMCT11, *n* = 7; αPD1, *n* = 7; combo, *n* = 6). The data represent three independent experiments. The error bars indicate the mean ± the standard error of the mean. The statistical analysis was performed by a two-way ANOVA with Šidák’s multiple comparison for **b**–**f** or by a two-way ANOVA with Dunnett’s multiple comparison test for **g** and **h**. Panel **a** was created with BioRender.com. Source data

**Extended Data Fig. 9:**
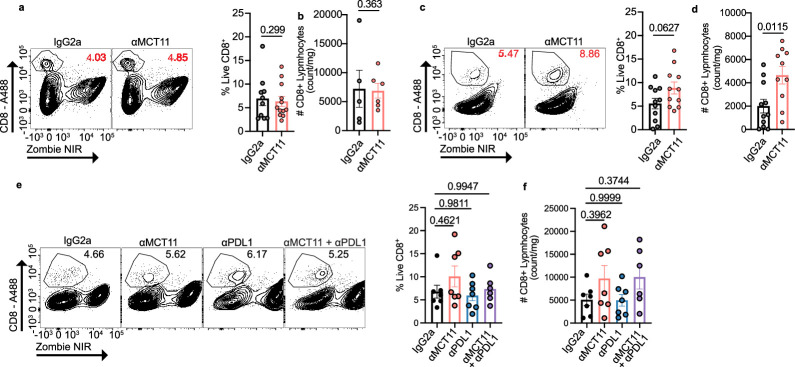
Effects of MCT11 therapy on total CD8 + T cells in tumors. **a)** Representative flow cytometry plot and quantification of percentage of live CD8^+^ T cells infiltrating B16 when treated with isotype (n = 11) or αMCT11 (n = 12). **b)** Total counts of CD8^+^ T cells infiltrating B16 when treated with isotype (n = 5) or αMCT11 (n = 6). **c)** Representative flow cytometry plot and quantification of percentage and **d**) total counts of live CD8^+^ T cells infiltrating MEER when treated with isotype control (n = 11) or αMCT11 (n = 11). **e)** Representative flow cytometry plot and quantification of percentage and **f)** total counts of live CD8^+^ T cells infiltrating MC38 when treated with isotype control (n = 7), αMCT11 (n = 7), αPD1 (n = 7) or αMCT11 + αPD1 (n = 6). Data represent two (**a**-**b**), or three (**c**-**f**) independent experiments. Error bars indicate +/− SEM. Statistical analysis performed by unpaired two-tailed Student’s T tests (**a**-**b**) or by one-way ANOVA with Tukey’s multiple comparison (**c**-**f**). Source data

To further understand effects of αMCT11 antibody blockade on T_ex_ cells in vivo, tumor-bearing mice were treated with three doses of αMCT11 therapy, and tumor infiltrates were analyzed on day 14 (200 μg per dose) (Fig. 5a). MCT11 blockade led to minimal changes in CD8^+^ T cell infiltration (Extended Data Fig. 9a,b), coinhibitory marker expression and cytokine production in B16 tumor infiltrates (Fig. 5b,c), which was not surprising given the relatively minor effect of αMCT11 therapy on B16 tumor burden. We then examined the infiltrate in MEER, given its particularly robust response to MCT11 blockade, at a timepoint before the tumor sizes diverged. The blockade of MCT11 in MEER-bearing mice resulted in increased total CD8^+^ T cells infiltrating the tumor (Extended Data Fig. 9c,d), no numerical change in the CD8^+^ TIL subpopulations (Fig. 5d) and increased CD8^+^ polyfunctionality specifically in the exhausted subpopulation (Fig. 5e). Similar to MCT11 conditional knockout animals, we found that MCT11 therapy in MEER tumors led to a decrease in hypoxia experienced by T_ex_ cells, while not affecting T_pex_, suggesting that MCT11 blockade can redirect T_ex_ cells to nonhypoxic regions of tumors (Fig. 5f). Finally, we wanted to test the effect of αMCT11/αPD1 combination therapy on MC38-infiltrating CD8^+^ T cells. While no effect was observed on the number of total or subset infiltrating CD8^+^ TIL (Extended Data Fig. 9e,f and Fig. 5g), we observed selective increases in T_ex_ cell polyfunctionality (Fig. 5h). Taken together, these data suggest blockade of MCT11 on T_ex_ cells alters their function to support superior antitumor immune responses.

## Discussion

Effective treatment of cancer with immunotherapy can be limited by the cellular and metabolic makeup of the TME. T cell exhaustion, a persistent dysfunctional fate acquired through altered signals and metabolic stress, renders CD8^+^ T cells unable to respond to stimulation effectively. Studies from our group and others implicate metabolic and mitochondrial dysfunction as major drivers of differentiation to the dysfunctional state of T_ex_ cells^8,9,18,42^. However, how the metabolic microenvironment may actively enforce dysfunction in these cells remains unclear.

Our data highlight MCT11 as a novel transporter expressed on the surface of T_ex_ cells (Fig. 1). We show *Slc16a11* is poised for transcription in T_ex_ cells and is driven to expression by tissue specific factors such as hypoxia (Fig. 2). The *Slc16a11* gene locus is bound and activated by TOX and BATF (Fig. 2e,f) and that deletion of HIF1α reduces the expression of MCT11 (Fig. 2l). It is possible other transcription factors are involved in driving the expression of MCT11 in T_ex_ cells. Commitment to exhaustion is a complex process involving large networks of TFs, such as T-bet, eomesodermin, Blimp1 and NFAT^43–45^. Therefore, in addition to TOX and BATF, several other TFs associated with exhaustion could be involved in driving MCT11 expression, although we suspect TFs promoted by tissue cues may be dominant in stabilizing MCT11 expression.

We also show MCT11 expression sensitizes T_ex_ cells to the TME, promoting the influx of lactic acid in tumor-infiltrating T_ex_ cells, limiting their effector functions (Fig. 3). Notably, conditional deletion of MCT11 in T_ex_ cells reduced lactic acid metabolism nearly to the level of T_pex_ cells (Fig. 1m). This caused the retention of a progenitor-like transcriptional program in T_ex_ cells, with increased *Tcf7* and *Myb* expression, TFs shown to promote response to αPD1 therapy^4,46^. Therefore, MCT11 deficient mice were primed for a superior response to αPD1 therapy (Fig. 3h–k).

T_ex_ cells are characterized by upregulating multiple coinhibitory receptors, but these cells also express high levels of costimulatory molecules, cytokine receptors and, as we’ve demonstrated here, a unique set of nutrient transporters. While ICBs have been a groundbreaking success in the clinic, only ~20% of patients with cancer respond to immunotherapy^47^. Identifying new targets for immunotherapy has been a challenge due to redundancy in checkpoint receptors and potential autoimmune side effects blocking inhibitory pathways. Importantly, MCT11 blockade acts on a distinct pathway than traditional ICBs, as MCT11 blockade interferes with a metabolic process as opposed to blocking the repression of T cell activation. MCT11 antibody blockade has an effect as a monotherapy, leading to complete responses in MC38 and MEER models and also as a combination therapy, doubling the CRs in mice bearing MC38 tumors in combination with αPD1 (Fig. 4e,f). MCT11 offers an exciting novel target given its unique expression in tumor-infiltrating PD1^hi^ and T_ex_ CD8^+^ T cells (Fig. 1l and Extended Data Fig. 4). Further, according the NCBI tissues gene atlas, MCT11 expression is limited (mean RPKM of ~1) and restricted to small populations of cells in the liver, lung and kidney. Short-term targeting of MCT11 in patients with cancer could, thus, limit the possibilities of potential toxicities and adverse autoimmune reactions. Supporting this is that a germline-knockout mouse^28^ and our own pan-tissue conditional knockout animal have no overt phenotypes.

Lactic acid has long been associated with immunosuppression, and we and others have discussed how lactic acid buildup in cancer can promote an environment of immunosuppression by directly inhibiting T cells^12,13^ and by stabilizing regulatory T cells^30^. However, lactate may not be universally suppressive; recent studies have shown that lactate is an essential carbon source for the TCA cycle in CD8^+^ T cells^48^. Nevertheless, concentration is important, and lactic acid is present at up to ten times higher in the TME than in serum^31^. In addition, lactic acid extruded by cancer cells acidifies the TME, which can have inhibitory effects on CD8^+^ T cell antigen recognition, cytokine production and cytotoxicity^12,13^. While T cells utilize lactate to fuel the TCA cycle, hypoxia reduces the ability to engage OXPHOS, and it is possible that a large influx of monocarboxylates through MCT11 could lead to inefficient buildup and storage of carbon, stabilization of lactate-sensitive transcription factors or alterations to the epigenome. In support of this idea, a recent study showed that tumor-infiltrating CD8^+^ T cells had an increase in intracellular accumulation of lactic acid, which was not being efficiently shuttled into the mitochondria and led to increased histone lysine lactylation^49^. Further, signals such as persistent TCR stimulation, inflammation and checkpoint receptor signaling may alter the ways in which lactate may be metabolized by cells. Further, while this study focuses on lactic acid, we do not discount other monocarboxylates present in the TME, albeit at much lower concentrations than lactic acid, as substrates for MCT11. Members of the MCT family can transport multiple monocarboxylates^41^ such as pyruvate^27^, acetate, succinate and ketone bodies.

Our study supports an evolving model of CD8^+^ T_ex_ cells being essentially hypersensitive to their extracellular milieu, both in costimulatory/inhibitory receptors and also nutrients. They upregulate MCT11, which renders them sensitive to lactic acid present at high levels in the TME. Teleologically, MCT11 may be expressed in CD8^+^ T cells to export lactic acid inside of highly metabolically active cells, or it may have evolved to allow tissues (which can accumulate lactate as a consequence of inflammation) to shape inflammatory function in persistently stimulated T cells. Our study highlights how immunity can be shaped by the local metabolic environment, such that over the course of differentiation, cells dynamically alter their nutrient transporter expression, rendering them differentially sensitive to metabolites in various tissues. By modulating nutrient transporters, such as in the case of MCT11, terminally differentiated T cells can be rendered insensitive to metabolites such as lactic acid, driving tumor eradication and therapeutic response.

## Methods

### Tumor cell lines

We obtained B16-F10 (CRL-6475) and A549 (CCL-185) cells from the American Type Culture Collection. We obtained MC38 (available at Kerafast) cells from D.A.A. Vignali (University of Pittsburgh) and MEER cells from R. Ferris (University of North Carolina). The B16, A549 and MC38 cell lines were authenticated via sequencing by their supplier. MEER cells were generated by overexpressing E6/E7 and Ras in primary mouse tonsil epithelial cells^50^ and were probed by western blot to confirm E6/E7 and Ras overexpression. MC38 and MEER were confirmed mycoplasma free in 2016 and B16 in 2018. Primary and immortalized cell lines were maintained in laboratory-made R10 medium (RPMI 1640, 10% fetal bovine serum, 2 mM ʟ-glutamine, penicillin plus streptomycin, nonessential amino acids, 1 mM sodium pyruvate, 5 mM HEPES buffer and β-mercaptoethanol). The cultures were incubated in temperature-stable and partial-pressure-stable conditions at 37 °C and 5% CO_2_.

### Mice

The work with mice was done in accordance with the Institutional Animal Care and Use Committee at the University of Pittsburgh. The mice were housed on a 12 h light–dark cycle in boxes (either four males or five females per box), in specific pathogen-free conditions. All experimental mice were fed with Purina Prolab Isopro RMH 3000 (5P75 and 5P76) chow ad libitum. Male and female mice were used on a C57BL/6 background between the ages of 8 and 12 weeks. *Slc16a11*^f/f^ mice were generated by S. Gingras (University of Pittsburgh). NSG, C57BL/6, SJ/L (Thy1.1), CMV^Cre^, *Cd4*^*cre*^ and Tg(TcraTcrb)1100Mjb/J (OT-I) mice were obtained from the Jackson Laboratory.

### Tumor growth curves and therapies

*Slc16a11*^f/f^ or *Slc16a11*^f/f^xCD4^Cre^ mice were injected with B16 100,000 cells in RPMI medium and treated with 200 μg per dose of αPD1 (Bio X Cell; clone RMP1-14; catalog number BE0146) for three total doses. For αMCT11-treated tumor growth curves, C57BL/6 or *Rag2*-knockout mice were injected with 250,000 cells of either B16, MC38 or MEER in serum-free RPMI. Starting when tumors were palpable (day 5–7) mice were treated with 200 μg per dose of mIgG2a isotype control (Bio X Cell), αPD1 (Bio X Cell; clone J43; catalog number BE0033-2), αMCT11 or LALAPG Fc mut αMCT11, which were administered every other day for a total of five doses. Anti-MCT11 was originally raised in mice against a human N-terminal peptide, and the monoclonal parental or LALAPG anti-MCT11 used in this study was generated recombinantly in CHO cells (Evitria). The tumors were measured three times per week using digital calipers. The maximal tumor size was reached when a tumor grew to 15 mm in size in any direction, at which point the tumor-bearing mouse was killed. The mice were excluded from analysis when tumors became ulcerated before the tumor reached maximal size.

### Tumor and lymph node collection and mechanical disruption

For T cell single-cell suspension, LNs and spleens of mice were mechanically disrupted with the back end of a syringe plunger and filtered through 70 μm filters (Fisher brand). For tumor single-cell suspensions, whole tumors were injected with 2 mg ml^−1^ of collagenase type IV, 2 U ml^−1^ of dispase and 10 U ml^−1^ of DNAse I (Sigma) in buffered RPMI and incubated for 20 min at 37 °C. The tumors were then mechanically disrupted using the back end of a syringe plunger and filtered through 70 μm filters (Fisher brand).

### LCMV infections

C57BL/6 mice were inoculated with LCMV clone 13 (2 × 10^6^ plaque forming units (PFU) by retro-orbital injection). The mice were monitored for weight loss to determine whether they were infected. The mice were sacrificed on day 14 post infection and their LNs, spleens, liver, kidney, lung and BM were collected. The organs were mechanically disrupted as described above to reach a single-cell suspension. Antigen specific T_ex_ cells were identified using a gp33^+^ tetramer gifted by Larry Kane (University of Pittsburgh).

### CD8^+^ T cell negative selection

CD8^+^ T cells were purified by negative selection from LNs and tumor single-cell suspensions. These were performed using Mojosort magnetic-beads (BioLegend) and the following biotinylated antibodies (BioLegend): CD4 (RM4-5, catalog number 100508, lot number B286276, dilution 1:1,000), CD19 (6D5, catalog number 115504, lot number B353713, dilution 1:1,000), CD11c (N418, catalog number 117304, lot number B317309, dilution 1:1,000), CD11b (M1/70, catalog number 101204, lot number B307868, dilution 1:1,000), Ly6G/Ly6C (Gr-1, catalog number 108404, lot number B351067, dilution 1:1,000), TCRγδ (GL3, catalog number 118103, lot number B355058, dilution 1:1,000), B220 (RA3-6B2, catalog number 103204, lot number B352779, dilution 1:500), CD49 (DX5, catalog number 108904, lot number B285502, dilution 1:500), CD105 (MJ7/18, catalog number 120404, lot number B266720, dilution 1:500), CD24 (M1/69, catalog number 101803, lot number B360781, dilution 1:500) and CD16/32 (93, catalog number 101303, lot number B355428, dilution 1:500).

### Hypoxia detection with pimonidazole

For experiments using Hypoxyprobe, the mice were retro-orbitally injected with pimonidazole (80 mg kg^−1^, Hypoxyprobe) in PBS 1 h before they were killed. Pimonidazole was detected using antipimonidazole antibodies (Hypoxyprobe) after 10 min of 4% paraformaldehyde (PFA) fixation, followed by Foxp3 Fix/Perm permeabilization for 20 min.

### Flow sorting and cytometry

For extracellular stains, the samples were incubated on ice for 20 min in an antibody cocktail mix. For intracellular stains, the samples were incubated on ice for 20 min in an antibody cocktail mix after fixation. The antibodies were obtained from the following companies: BioLegend: anti-CD4 (GK1.5, catalog number 100412, lot number B184560, dilution 1:1,000), anti-CD8 (53-6.7, catalog number 100707, lot number B171971, dilution 1:1,000), anti-CD44 (IM7, catalog number 103032, lot number B267976, dilution 1:500), CD45 (I3/2.3, catalog number 147711, lot number B254856, dilution 1:1,000) anti-CD147 (OX-114, catalog number 123716, lot number B262975, dilution 1:500), anti-CD19 (6D5, catalog number 115530, lot number B276004, dilution 1:1,000), anti-Ly6G (1A8, catalog number 127616, lot number B248844, dilution 1:500), anti-Ly6C (HK1.4, catalog number 128017, lot number B213757, dilution 1:500), anti-MHCII (M5/114.15.2, catalog number 107612, lot number B251993, dilution 1:500), anti-F4 80 (BM8, catalog number 123149, lot number B326894, dilution 1:250), anti-CD279 (PD1, 29F.1A12, catalog number 135221, lot number B194160, dilution 1:250), anti-HAVcr-2 (TIM3, RMT3-23, catalog number 119705, lot number B224472, dilution 1:250), anti-IFNγ (XMG1.2, catalog number 505842, lot number B270630, dilution 1:250), anti-TNF (MP6-XT22, catalog number 506322, lot number B218553, dilution 1:500), anti-CD11b (M1/70, catalog number 101204, lot number B307868, dilution 1:250) and anti-CD11c (N418, catalog number 117320, lot number B286499, dilution 1:250); Invitrogen: anti-CD62L (MEL-14, catalog number 564109, lot number 7341887, dilution 1:500), and anti-TOX (TXRX10, catalog number 80-6502-82, lot number 2246902, dilution 1:250). The human samples were stained with the following antibodies (BioLegend): anti-PD1 (EH12.2Z7, catalog number 329904, dilution 1:200), anti-TIM3 (F382E2, catalog number 345006, dilution 1:200), anti-CD8 (HIT8a, catalog number 300918, dilution 1:200) and anti-CD3 (SK7, catalog number 344834, dilution 1:200). For panels targeting transcription factors, the cells were fixed with the FoxP3 fix/perm buffer set (BioLegend) according to the manufacturer’s protocol. For panels targeting cytokines, fixation was performed with Cytofix/Cytoperm (BD Biosciences) according to the manufacturers protocol. The data collection utilized BD FACSDiva v9.0 for flow cytometry and was analyzed via FlowjoV10.

### CD8^+^ TIL cytokine production assay

Tumor and lymph node cell suspensions were stimulated in complete R10 medium with Golgi-Plug (BD Biosciences), 3 μg ml^−1^ plate-bound αCD3 and 2 μg ml^−1^ αCD28 in complete R10 medium with Golgi-Plug for 5 h at 37 °C. In addition, dLNs and tumor cells were cultured in complete R10 medium Golgi-Plug, as a no stimulus control to determine gating strategies.

### CRISPR–Cas9 knockout

The CRISPR–Cas9-mediated knockout method was modified on the basis of a previous publication. A total of 10 µg of Alt-R S.p. Cas9 Nuclease V3 (IDT) was mixed with an LDHa-targeted sgRNA (AAGCTGGTCATTATCACCGC) to form an RNP complex. A total of 2 million MEER cells were mixed with the RNP and Lonza SF buffer along with the Alt-R Cas9 Electroporation Enhancer for electroporation. The DJ-110 program (Lonza 4D Nucleofector) was used for electroporation.

### Tumor interstitial fluid lactate measurement

The tumors were collected from mice and measured for tumor weight. The tumors were then cut up and placed on 20 mm nylon filters (Spectrum labs) and placed in top of 50 ml conical tube, wedged between the cap and the filter. The conical tube was then spun for 5 min at 2,000*g*. Approximately 20 μl of tumor interstitial fluid was then collected from the bottom of the conical tube. To measure lactate concentrations, we utilized a lactate meter (Nova Biomdeicals). The lactate concentration was then divided by tumor weight to generate final concentration of lactate.

### T cell transduction, retroviral overexpression of MCT11 and adoptive transfer

The *Slc16a11* murine coding sequence was obtained from the National Institutes of Health (NIH) database and cloned via Gibson assembly into a murine stem cell virus retroviral expression vector, which also encodes for an internal ribosome entry site–mCherry cassette. The vector was transfected into the Plat-E retroviral packaging cell line. The CD8^+^ T cells were collected and negatively isolated with Mojo beads (as described above) and were stimulated for 24 h with 5 μg ml^−1^ of αCD3, 2 μg ml^−1^ of αCD28 and 50 U ml^−1^ of IL-2. After 48 h, Plat-E retroviral supernatant was collected and supplemented with 5 μg ml^−1^ polybrene. The OT-I T cells were spun down and transduced in the viral supernatant for 2 h at 2,000 rpm. The cells were expanded and sorted via mCherry fluorescence on day 3 post transduction. A total of 3 × 10^6^ OT-I T cells were then retro-orbitally transferred into D7 B16^OVA^-bearing mice.

### Human CAR-T cell production and adoptive transfer

Human CD8^+^ T cells were isolated from bulk PBMCs from the blood bank. The T cells were then stimulated with anti-CD3/CD28 Dynabeads in RPMI supplemented with 10% fetal bovine serum (v/v) and 200 U ml^−1^ human IL-2 at 37 °C with 5% CO_2_ for 48 h. Then, CD19-CAR-expressing retrovirus was added to the expanding cells. After 5 days of expansion, Dynabeads were magnetically removed, and the cells were expanded for another 5 days in the presence of IL-2. CD19-A549-bearing NSG mice were adoptively transferred with 3 × 10^6^ human CD8^+^ T cells expressing CD19-CAR.

### Transcriptomic analysis by RNA-seq

RNA-seq data of B16 melanoma CD8^+^ TIL populations^21^ was utilized to evaluate expression of SLCs (GSE175408). For sequencing of the MCT11 conditional knockout T cells, 350 T_ex_ cells were sorted from B16 tumors into lysis buffer in a 96-well plate. For all samples, complementary DNA was generated using the SMARTer Ultra Low Input RNA Kit for sequencing. The libraries were generated using the Nextera XT kit (Illumina) with 1 ng of cDNA in a total of 5 μl. Sequencing was done using a P3 flow cell-NextSeq2000. Bulk RNA-seq analysis was performed on PartekFlow. The paired-end reads were concatenated into a single fastq file. The reads were trimmed for adapters using Cutadaptv1.12 before being aligned to *Mus musculus* reference genome (mm38) using the RNA-seq aligner STAR2.7. Using the raw counts, differential genes were found by DESeq2. Publicly available RNA-seq data^21^ from WT T_ex_ cells were used as controls (GSE175408). A gene set enrichment analyses of selected immunologic signature and hallmark gene sets was performed with clusterProfiler (10.1016/j.xinn.2021.100141).

### Transcriptomic analysis by single-cell RNA-seq

Single-cell RNA-seq data from blood and tumor-infiltrating immune populations from a cohort of patients with head and neck cancer^22,23^ was utilized to evaluate the expression of MCT11 across subsets of CD8^+^ T cells. Feature/barcode expression matrices were downloaded from the Gene Expression Omnibus (GSE139324), and cell type annotations were inferred as previously described^3,4^. CD8^+^ T cells were then bioinformatically isolated from other immune populations, and the top 2,000 highly variable genes were used as input for dimensionality reduction with principal component analysis. The top principal components were identified heuristically by identifying the inflection point on an elbow plot and were subsequently used for generating uniform manifold approximation and projection embeddings and Louvian-based clustering. The coexpression of *PDCD1*, *HAVCR2* and *SLC16A11* was then evaluated across clusters in PBMC and TIL and was visualized with a heat map.

### [^14^C]-lactic acid oxidation experiments

The protocol is modified from previous published research^51^. T cells were incubated for 6 h in 0.2 ml RPMI containing 3 mM uCi ml^−1^ [^14^C]-lactic acid, sodium salt (NEC599050UC, PerkinElmer) and 2.5 mM sodium lactate. Afterward, the medium was transferred to borosilicate glass tubes. Each glass tube contained a microcentrifuge tube filled with 1 N sodium hydroxide to absorb CO_2_. The glass tubes were then sealed, and 5 N hydrochloric acid was injected into each tube to stop cellular metabolism and release carbon dioxide. After overnight absorption of [^14^C]-CO_2_, the samples were analyzed by liquid scintillation counting. A total of 25,000 T cells were used for ex vivo TIL studies and 100,000 were used for MCT11 overexpressing cells.

### Continuous stimulation under hypoxia assay

The protocol was previously published^8^. Briefly, CD8^+^ T cells were isolated from mouse LNs or human PBMCs. The T cells were then activated at a 1:1 ratio with CD3/CD28 Dynabeads for 24 h. After 24 h, the T cells were split into four groups: acute TCR stimulus (no beads for remainder of assay in 20% oxygen), acute TCR stimulus under hypoxia (no beads for remainder of assay in 1.5% oxygen), continuous stimulus (10:1 beads to T cells for remainder of assay in 20% oxygen) and continuous stimulus under hypoxia (10:1 beads to T cells for remainder of assay in 1.5% oxygen). The t cells were kept in those conditions for 6 days (mouse assay) or 8 days (human assay) at a 1 million per milliliter concentration in R10 media with 50 IU IL-2.

### Extracellular flux analysis

In vitro cultured B16 and MEER tumor cell lines were plated on an Agilent Seahorse poly-d-lysine-coated cell culture plate at 25,000 cells per well in minimal RPMI supplemented with 2 mM glutamine. The basal glycolytic rates were measured for 30 min, following the injections of 10 mM glucose, 2 μM oligomycin and 10 mM 2-deoxyglucose, and the readings were continued for 3 h. The measurements were performed on the XFe96 Analyzer.

### Western blots

The tumor cells were lysed in RIPA lysis buffer with sodium orthovanadate and protease inhibitor for 15 min on ice. The lysates were spun down via high-speed centrifugation to clear debris and samples were mixed with 4× lithium dodecyl sulfate (LDS) buffer and boiled for 10 min. The lysates were loaded onto gels and ran at 200 V for 60 min. The proteins were transferred onto nitrocellulose membranes with transfer buffer at 30 V for 90 min. Blocking of the membrane was performed with nonfat dry milk for 1 h and washed with Tris-buffered saline + 0.1% Tween-20 (TBST). The membranes were washed three times with TBST and probed with a primary antibody overnight and horseradish peroxidase (HRP)-conjugated secondary antibody for 1 h at 4 °C. The membrane was then washed three times with TBST and incubated for 1 min with chemiluminescent substrate. The western blots were detected via chemiluminescent exposure to film. The antibodies used were b actin (Cell Signaling Technology) and LDHA (Cell Signaling Technology).

### Sample sizes, randomization and blinding

No statistical methods were used to predetermine sample sizes, but our sample sizes are similar to those reported in previous publications. The tumor growth curves were randomized on the basis of initial tumor size once tumors became palpable, ensuring even distribution across groups. All tumor growth curves were conducted in a blinded manner—one author administered treatments while another independently measured tumor size. For all other experiments, data collection and analysis were not performed blind to the conditions of the experiments.

### Statistical analysis

The data distribution was assumed to be normal, but this was not formally tested. We used unpaired or paired Student’s *t*-tests, one-way analysis of variance (ANOVA) with Tukey’s multiple comparisons or two-way ANOVA with Tukey’s, Dunnett’s or Šidák’s multiple comparison tests to calculate the *P* values in GraphPad Prism. For tumor growth curves and survival curves, the *P* values were calculated using two-way ANOVA with Tukey’s multiple comparison and a log rank Mendel–Cox test, respectively. Statistical tests for specific experiments can be found in the figure legends. Grubb’s test was utilized to determine whether a value was a significant statistical outlier.

### Reporting summary

Further information on research design is available in the Nature Portfolio Reporting Summary linked to this article.

## Online content

Any methods, additional references, Nature Portfolio reporting summaries, source data, extended data, supplementary information, acknowledgements, peer review information; details of author contributions and competing interests; and statements of data and code availability are available at 10.1038/s41590-024-01999-3.

## Supplementary information

Reporting Summary

## Source data

Source Data Fig. 1 Source data for Fig. 1.

Source Data Fig. 2 Source data for Fig. 2.

Source Data Fig. 3 Source data for Fig. 3.

Source Data Fig. 4 Source data for Fig. 4.

Source Data Fig. 5 Source data for Fig. 5.

Source Data Extended Data Fig. 2 Source data for Extended Data Fig. 2.

Source Data Extended Data Fig. 3 Source data for Extended Data Fig. 3.

Source Data Extended Data Fig. 4 Source data for Extended Data Fig. 4.

Source Data Extended Data Fig. 5 Source data for Extended Data Fig. 5.

Source Data Extended Data Fig. 6 Source data for Extended Data Fig. 6.

Source Data Extended Data Fig. 7 Source data for Extended Data Fig. 7.

Source Data Extended Data Fig. 8 Source data for Extended Data Fig. 8.

Source Data Extended Data Fig. 9 Source data for Extended Data Fig. 9.

Source Data Extended Data Fig. 8j This is the original unprocessed blot used in Extended Data Fig. 8j.

## Data Availability

RNA-seq data have been deposited in the Gene Expression Omnibus (GEO) under accession code GSE249944. Single-cell RNA-seq from the GEO repository are deposited under accession code GSE139324. Source data for Fig. 1a,b and Extended Data Fig. 2b, as well as WT control for Fig. 3g,h, are available in the GEO repository under accession code GSE175408.
