# Supplementary material for: Dysfunction of exhausted T cells is enforced by MCT11-mediated lactate metabolism
Source: Nat Immunol. 2024 Nov 8;25(12):2297–307. doi: 10.1038/s41590-024-01999-3 (PMC11588660; doi:10.1038/s41590-024-01999-3)
Supplement: Supplementary file 1 — Reporting Summary [file 41590_2024_1999_MOESM1_ESM.pdf]

nature portfolio

Corresponding author(s): Greg Delgoffe

Last updated by author(s): Sep 30, 2024

Reporting Summary

Nature Portfolio wishes to improve the reproducibility of the work that we publish. This form provides structure for consistency and transparency in reporting. For further information on Nature Portfolio policies, see our Editorial Policies and the Editorial Policy Checklist.

Statistics

For all statistical analyses, confirm that the following items are present in the figure legend, table legend, main text, or Methods section.

n/a	Confirmed	
☐	☒	The exact sample size (*n*) for each experimental group/condition, given as a discrete number and unit of measurement
☐	☒	A statement on whether measurements were taken from distinct samples or whether the same sample was measured repeatedly
☐	☒	The statistical test(s) used AND whether they are one- or two-sided<br>*Only common tests should be described solely by name; describe more complex techniques in the Methods section.*
☐	☒	A description of all covariates tested
☐	☒	A description of any assumptions or corrections, such as tests of normality and adjustment for multiple comparisons
☐	☒	A full description of the statistical parameters including central tendency (e.g. means) or other basic estimates (e.g. regression coefficient) AND variation (e.g. standard deviation) or associated estimates of uncertainty (e.g. confidence intervals)
☐	☒	For null hypothesis testing, the test statistic (e.g. *F*, *t*, *r*) with confidence intervals, effect sizes, degrees of freedom and *P* value noted<br>*Give P values as exact values whenever suitable.*
☒	☐	For Bayesian analysis, information on the choice of priors and Markov chain Monte Carlo settings
☒	☐	For hierarchical and complex designs, identification of the appropriate level for tests and full reporting of outcomes
☒	☐	Estimates of effect sizes (e.g. Cohen's *d*, Pearson's *r*), indicating how they were calculated

Our web collection on statistics for biologists contains articles on many of the points above.

Software and code

Policy information about availability of computer code

Data collection	Data collection utilized BD FACSDiva v9.0 for flow cytometry, Seahorse Wave Controller Software V2.6.3.8 for extracellular flux assays.
Data analysis	Data was analyzed with FlowJo V10 and Prism10. Bulk RNA-seq analysis was performed on PartekFlow. Paired end reads were concatenated into a single fastq file. Reads were trimmed for adapters using CutadaptV1.12 before being aligned to Mus musculus reference genome (mm38) using the RNA-seq aligner STAR2.7. Using the raw counts. differential genes were found by DESeq2. Gene set enrichment analyses of selected immunologic signature and hallmark gene sets was performed with clusterProfiler (REF https://doi.org/10.1016/j.xinn.2021.100141). Single-cell RNAseq data from blood and tumor infiltrating immune populations from a cohort of head and neck cancer patients was utilized to evaluate the expression of MCT11 across subsets of CDS+ T cells. Feature/barcode expression matrices were downloaded from the Gene Expression Omnibus and cell type annotations were inferred as previously described. CDS+ T cells were then bioinformatically isolated from other immune populations. and the top 2000 highly variable genes were used as input for dimensionality reduction with principal component analysis. The top principal components were identified heuristically by identifying the inflection point on an elbow plot and were subsequently used for generating uniform manifold approximation and projection (UMAP) embeddings and Louvian-based clustering. Co-expression of PDCD1, HAVCR2 and SLC16A 11 was then evaluated across clusters in PBMC and TIL and was visualized with a heatmap.

For manuscripts utilizing custom algorithms or software that are central to the research but not yet described in published literature, software must be made available to editors and reviewers. We strongly encourage code deposition in a community repository (e.g. GitHub). See the Nature Portfolio guidelines for submitting code & software for further information.

Data

Policy information about availability of data

All manuscripts must include a data availability statement. This statement should provide the following information, where applicable:

- Accession codes, unique identifiers, or web links for publicly available datasets
- A description of any restrictions on data availability
- For clinical datasets or third party data, please ensure that the statement adheres to our policy

RNA-seq data have been deposited to the Gene Expression Omnibus (GEO) with accession no. GSE249944. Source data for single cell RNA sequencing are available in the GEO repository, accession no. GSE139324. Source data for Figure 1a-b and Extended Figure 2b, as well as WT control for Figure 3g-h, are available in the GEO repository, accession no. GSE175408.

Research involving human participants, their data, or biological material

Policy information about studies with human participants or human data. See also policy information about sex, gender (identity/presentation), and sexual orientation and race, ethnicity and racism.

Reporting on sex and gender	N/A
Reporting on race, ethnicity, or other socially relevant groupings	N/A
Population characteristics	N/A
Recruitment	N/A
Ethics oversight	N/A

Note that full information on the approval of the study protocol must also be provided in the manuscript.

Field-specific reporting

Please select the one below that is the best fit for your research. If you are not sure, read the appropriate sections before making your selection.

☒ Life sciences ☐ Behavioural & social sciences ☐ Ecological, evolutionary & environmental sciences

For a reference copy of the document with all sections, see nature.com/documents/nr-reporting-summary-flat.pdf

Life sciences study design

All studies must disclose on these points even when the disclosure is negative.

Sample size	Sample sizes were not statistically predetermined but were chosen based on previous work in exhausted T cell and tumor immunology (Scharping et al 2016 Immunity, Menk et al 2018 J Exp Med, Scharping et al 2017 Cancer Immunol Res., Najjar et al 2019 Cancer Immunol Res.). These sample sizes are sufficient to allow for the determination of statistical significance between groups and minimized the number of animals or replicates needed for each experiment.
Data exclusions	For tumor implantation studies in mice, we excluded data if tumors ruptured and ulcerated prior to the completion of the experiment as this dramatically alters the tumor microenvironment and longitudinal growth. Statistical outliers were determines based on Grubb's outlier test.
Replication	Experiments were conducted at least two independent times (and in most cases, at least three independent times) with all attempts of replication being successful.
Randomization	Mice were placed into experimental groups and were randomized into treatment types. For experiments with mouse-derived cells, cells were split and each given the same treatment to generate paired data.
Blinding	All tumor growth curves were conducted in a blinded manner—one author administered treatments while another independently measured tumor size. For all other experiments, data collection and analysis were not performed blind to the conditions of the experiments.

Reporting for specific materials, systems and methods

We require information from authors about some types of materials, experimental systems and methods used in many studies. Here, indicate whether each material, system or method listed is relevant to your study. If you are not sure if a list item applies to your research, read the appropriate section before selecting a response.

Materials & experimental systems

n/a	Involved in the study
☐	☒ Antibodies
☐	☒ Eukaryotic cell lines
☒	☐ Palaeontology and archaeology
☐	☒ Animals and other organisms
☐	☒ Clinical data
☒	☐ Dual use research of concern
☒	☐ Plants

Methods

n/a	Involved in the study
☒	☐ ChIP-seq
☐	☒ Flow cytometry
☒	☐ MRI-based neuroimaging

Antibodies

Antibodies used

BioLegend: anti-CD4 (GK1 .5, catalogue number 100412, lot number B184560, dilution 1 :1,000), anti-CDS (53-6.7, catalogue number 100707, lot number B171971, dilution 1 :1,000), anti-CD44 (IM7, catalogue number 103032, lot number B267976, dilution 1 :500), CD45 (13/2.3, catalogue number 147711, lot number B254856, dilution 1:1000) anti-CD147 (OX-114, catalogue number 123716, lot number B262975, dilution 1 :500), anti-CD19 (6D5, catalogue number 115530, lot number B276004, dilution 1:1000), anti-Ly6G (1 AB, catalogue number 127616, lot number B248844, dilution 1 :500), anti-Ly6C (HK1 .4, Catalogue number 128017, lot number B213757, dilution 1 :500), anti-MHCII (M5/114.15.2, catalogue number 107612, lot number B251993, dilution 1 :500), anti-F4 80 (8MB, catalogue number 123149, lot number B326894, dilution 1 :250), anti-CD279 (PD1, 29F.1A12, catalogue number 135221, lot number B194160, dilution 1 :250), anti-Tim3 (RMT3-23, catalogue number 119705, lot number B224472, dilution 1 :250), anti-lFNy (XMG1 .2, catalogue number 505842, lot number B270630, dilution 1 :250), anti-TNFa (MP6-XT22, catalogue number 506322, lot number B218553, dilution 1 :500), anti-CD11 b (M1/70, catalogue number 101204, lot number B307868, dilution 1 :250), and anti-CD11c (N418, catalogue number 117320, lot number B286499, dilution 1 :250).

Human samples were stained with the following antibodies (BioLegend): anti-PD1 (EH12.2Z7, catalogue number 329904, dilution 1 :200), anti-TIM3 (F382E2, catalogue number 345006, dilution 1 :200), anti-CDS (HIT8a, catalogue number 300918, dilution 1 :200), and anti-CD3 (SK7, catalogue number 344834, dilution 1 :200).

lnvitrogen: anti-CD62L (MEL-14, catalogue number 564109, lot number 7341887, dilution 1 :500), and anti-TOX (TXRX10, catalogue number 80-6502-82, lot number 2246902, dilution 1 :250).

Cell signaling: anti-LDHA (catalogue number 2012s, dilution 1:1000)

anti- PD1 (Bio X Cell; Clone RMP1-14; Catalogue:BE0146) &(Bio X Cell; Clone J43; Catalogue:BE0033-2)

MCT11 antibody

Validation

All antibodies and stains are commercially available and were validated by the manufacturer. MCT11 antibody was validated by staining MCT11 overexpressing cells.

Eukaryotic cell lines

Policy information about cell lines and Sex and Gender in Research

Cell line source(s)

We obtained B16-F10 (CRL-6475) and A549 (CCL-185) cells were obtained from the American Type Culture Collection (ATCC). MC38 cells were obtained from Dario Vignali (commercially available from Kerafast).MEER cells were obtained from Robert Ferris (originally from Jung, Y.-S. et al. CD200: association with cancer stem cell features and response to chemoradiation in head and neck squamous cell carcinoma. Head Neck 37, 327-335 (2015).)

Authentication

The B16, A549 cell line were authenticated in 2018 through independent sequencing by the supplier. The MC38 cell lines was authenticated in 2016 via sequencing by their supplier. MEER cells were generated by overexpressing E6/E7 and Ras in primary mouse tonsil epithelial cells (MTECs), and1 and were authenticated by the supplier in 2013 via western blot to confirm E6/E7 and Ras overexpression.

Mycoplasma contamination

MC38 and MEER were confirmed mycoplasma free in 2016, and A549 and B16 in 2018.

Commonly misidentified lines
(See ICLAC register)

No commonly misidentified cell lines were used in this study.

Animals and other research organisms

Policy information about studies involving animals; ARRIVE guidelines recommended for reporting animal research, and Sex and Gender in Research

Laboratory animals

C57BL/6, -Rag1-/-, CD4cre, OT-I, NSG and -Thy1 a (Thy1 .1 congenic) were purchased from The Jackson Laboratory. Slc16a11f/f were generated by Sebastien Gingras. All mice were housed in specific pathogen free conditions at an ambient temperature of 20-26 C and humidity of 30-70% with a 12:12 hour light dark cycle prior to use. The maximal tumor size of 15mm in any direction was not

	exceeded in any experiment. Both male and female mice were used in these studies. Mice used were between the age of six (6) and ten (10) weeks.
Wild animals	N/A
Reporting on sex	Both male and female mice were used in this study
Field-collected samples	N/A
Ethics oversight	Animal housing and studies were done in accordance with the Instituional Animal Care and Use Committee of the University of Pittsburgh (Protocol #23073380)

Note that full information on the approval of the study protocol must also be provided in the manuscript.

Clinical data

Policy information about clinical studies

All manuscripts should comply with the ICMJE guidelines for publication of clinical research and a completed CONSORT checklist must be included with all submissions.

Clinical trial registration	*Provide the trial registration number from ClinicalTrials.gov or an equivalent agency.*
Study protocol	*Note where the full trial protocol can be accessed OR if not available, explain why.*
Data collection	*Describe the settings and locales of data collection, noting the time periods of recruitment and data collection.*
Outcomes	*Describe how you pre-defined primary and secondary outcome measures and how you assessed these measures.*

Plants

Seed stocks	N/A
Novel plant genotypes	N/A
Authentication	N/A

Flow Cytometry

Plots

Confirm that:

- [x] The axis labels state the marker and fluorochrome used (e.g. CD4-FITC).
- [x] The axis scales are clearly visible. Include numbers along axes only for bottom left plot of group (a 'group' is an analysis of identical markers).
- [x] All plots are contour plots with outliers or pseudocolor plots.
- [x] A numerical value for number of cells or percentage (with statistics) is provided.

Methodology

Sample preparation	For T cell single cell suspension, lymph nodes and spleens of mice were mechanically disrupted with the back of a suringe plunger and filtered through a 70 micron filter (Fisher Brand). For tumor single cell suspensions, whole tumors were injected with 2mg/mL of collagenase type IV, 2U/mL of dispase and 10U/mL of DNAse I (Sigma) in buffered RPMI, and incubated for 20 minutes at 37 degrees Celsius. Tumors were then mechanically disrupted using the back of a syringe plunger and filtered through a 70 micron filter.
Instrument	BD Fortessa
Software	Flowjo V10
Cell population abundance	N/A
Gating strategy	Lymphocytes were gated using FSC-Area vs. SSC-Area. Doublet exclusion was done through comparison of SSC-Width to SSC-

Gating strategy	Area and FSC-Width to FSC-Area. Viable cells were determined with zombie dye, in which cells negative for zombie stain were viable. See Extended Data Figure 1 for detailed gating strategy.

☒ Tick this box to confirm that a figure exemplifying the gating strategy is provided in the Supplementary Information.
