# Supplementary figures and images for: Dysfunction of exhausted T cells is enforced by MCT11-mediated lactate metabolism
Source: Nat Immunol. 2024 Nov 8;25(12):2297–307. doi: 10.1038/s41590-024-01999-3 (PMC11588660; doi:10.1038/s41590-024-01999-3)

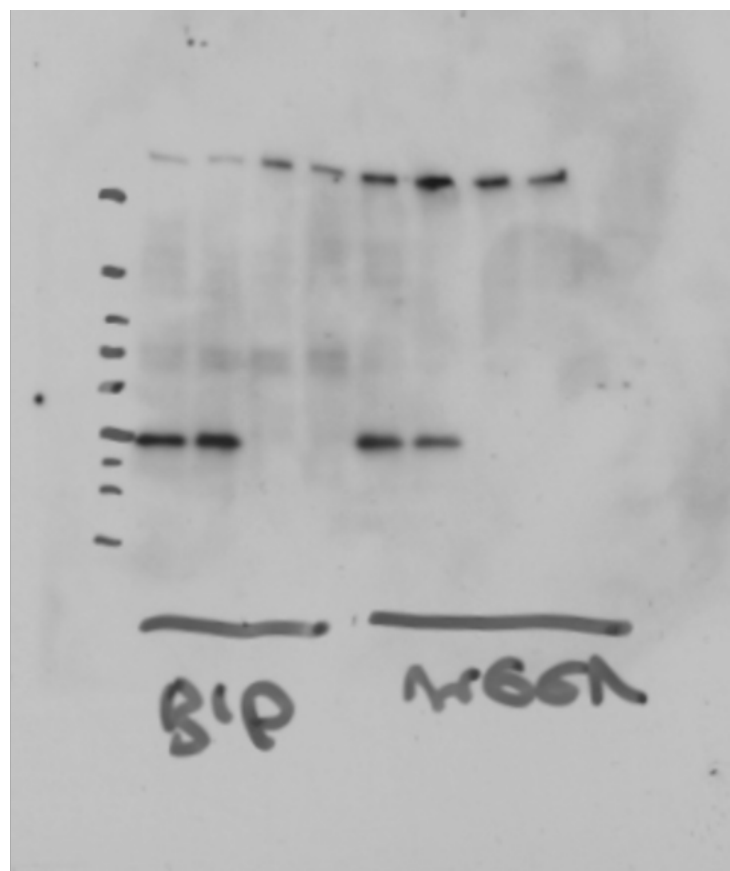

Supplement: Supplementary file 15 — This is the original unprocessed blot used in Extended Data Fig. 8j. [file 41590_2024_1999_MOESM15_ESM.pdf]
